# An Explainable Graph Neural Framework to Identify Cancer‐Associated Intratumoral Microbial Communities

**DOI:** 10.1002/advs.202403393

**Published:** 2024-09-03

**Authors:** Zhaoqian Liu, Yuhan Sun, Yingjie Li, Anjun Ma, Nyelia F. Willaims, Shiva Jahanbahkshi, Rebecca Hoyd, Xiaoying Wang, Shiqi Zhang, Jiangjiang Zhu, Dong Xu, Daniel Spakowicz, Qin Ma, Bingqiang Liu

**Affiliations:** ^1^ School of Mathematics Shandong University Jinan Shandong 250100 China; ^2^ College of Sciences Xi'an University of Science and Technology Xi'an Shanxi 710054 China; ^3^ Department of Biomedical Informatics The Ohio State University Columbus OH 43210 USA; ^4^ Pelotonia Institute for Immuno‐Oncology The Ohio State University Columbus OH 43210 USA; ^5^ Department of Internal Medicine College of Medicine The Ohio State University Columbus OH 43210 USA; ^6^ Department of Food Science and Technology College of Food Agricultural, and Environmental Sciences The Ohio State University Columbus OH 43210 USA; ^7^ Department of Human Sciences College of Education and Human Ecology The Ohio State University Columbus OH 43210 USA; ^8^ Department of Electrical Engineering and Computer Science University of Missouri Columbia MO 65201 USA; ^9^ Christopher S. Bond Life Sciences Center University of Missouri Columbia MO 65201 USA; ^10^ Shandong National Center for Applied Mathematics Jinan Shandong 250199 China

**Keywords:** attention mechanism, graph neural network, heterogeneous graph representation, intratumoral microbiome

## Abstract

Microbes are extensively present among various cancer tissues and play critical roles in carcinogenesis and treatment responses. However, the underlying relationships between intratumoral microbes and tumors remain poorly understood. Here, a MIcrobial Cancer‐association Analysis using a Heterogeneous graph transformer (MICAH) to identify intratumoral cancer‐associated microbial communities is presented. MICAH integrates metabolic and phylogenetic relationships among microbes into a heterogeneous graph representation. It uses a graph transformer to holistically capture relationships between intratumoral microbes and cancer tissues, which improves the explainability of the associations between identified microbial communities and cancers. MICAH is applied to intratumoral bacterial data across 5 cancer types and 5 fungi datasets, and its generalizability and reproducibility are demonstrated. After experimentally testing a representative observation using a mouse model of tumor‐microbe‐immune interactions, a result consistent with MICAH's identified relationship is observed. Source tracking analysis reveals that the primary known contributor to a cancer‐associated microbial community is the organs affected by the type of cancer. Overall, this graph neural network framework refines the number of microbes that can be used for follow‐up experimental validation from thousands to tens, thereby helping to accelerate the understanding of the relationship between tumors and intratumoral microbiomes.

## Graphical abstract

MICAH utilizes a heterogeneous graph transformer to uncover cancer‐related microbial communities within tumors. By integrating metabolic and phylogenetic data with transcriptomic data, it enhances the understanding of microbe‐cancer interactions. Tested on multiple cancer and fungi datasets, it identifies microbes for further cancer research, with experimentally validated bacteria showing increased anti‐PD1 therapy efficacy in a mouse model.

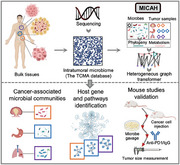

## 1 Introduction

Microbes have been detected as an intrinsic component of the tumor microenvironment across multiple cancer types.^[^ ^1^, ^2^, ^3^ ^]^ These microbes may promote tumor growth or stimulate the immune system to attack cancer cells.^[^ ^4^, ^5^ ^]^ Some evidence suggests that each cancer type has a unique microbiome, characterized by specific microbial communities.^[^ ^6^ ^]^ These microbial communities have been associated with cancer initiation, progression, and pathogenesis.^[^ ^2^, ^6^, ^7^, ^8^, ^9^ ^]^ Characterization of these cancer‐associated microbial communities holds the promise of unraveling the mechanistic links between intratumoral microbiomes and cancer. Moreover, such insights will shed light on translating microbiome research into clinical applications, particularly in manipulating intratumoral microbiomes to enhance cancer diagnosis and treatment.^[^ ^8^ ^]^

Investigating the links between the intratumoral microbiome and cancer tissues is incomplete due to the intratumoral microbiome's low biomass and decontamination difficulties.^[^ ^10^ ^]^ Fortunately, large‐scale human tissue sequencing data repositories, such as The Cancer Genome Atlas (TCGA) and the Oncology Research Information Exchange Network, are available resources for discovery.^[^ ^11^, ^12^ ^]^ Several computational tools, such as PathSeq and SRSA,^[^ ^13^, ^14^, ^15^, ^16^ ^]^ have been proposed to mine intratumoral microbial reads from existing tissue sequencing data, which enables the characterization of the tissue‐resident metagenome.^[^ ^1^, ^10^, ^17^, ^18^ ^]^ The resulting data, such as The Pan‐Cancer Mycobiome Atlas and The Cancer Microbiome Atlas (TCMA),^[^ ^1^, ^10^ ^]^ serve as valuable resources for exploring the intratumoral microbiome.

Existing approaches for identifying disease‐associated gut microbiomes can be extended to investigate cancer‐associated intratumoral microbiomes.^[^ ^19^, ^20^ ^]^ The most commonly used methods rely on statistical analyses to detect individual microbes with significant differential abundance between different groups,^[^ ^21^, ^22^, ^23^, ^24^, ^25^, ^26^, ^27^ ^]^ such as corncob,^[^ ^23^ ^]^ LEfSe,^[^ ^25^ ^]^ and DESeq2.^[^ ^27^ ^]^ However, a crucial limitation of these approaches is that they overlook the inherent relationships among microbes within a community. To address this limitation, some studies have attempted to construct co‐occurrence networks to explore the associations between various microbial species^[^ ^28^, ^29^ ^]^ and perform network structure comparisons between different populations to evaluate the associations of microbes with diseases. Nevertheless, creating reliable networks representing the true species‐species interactions remains challenging due to microbial data's sparsity and high dimensionality.^[^ ^30^, ^31^ ^]^ Furthermore, these approaches are confined to comparing single pairs of networks, potentially introducing bias and limiting their effectiveness in downstream analysis.

Recognizing the potential of machine learning models to extract valuable insights from complex data, researchers have applied both traditional machine learning methods^[^ ^32^, ^33^ ^]^ like Random Forest (RF) and least absolute shrinkage and selection operator (Lasso), as well as deep learning approaches like MIIDL and PopPhy‐CNN,^[^ ^34^, ^35^ ^]^ for disease‐associated microbiome identification. These methods typically address the question by framing it as a host phenotype classification task, followed by feature selection to identify disease‐associated microbes. While they have demonstrated considerable success in host phenotype classification,^[^ ^34^, ^35^ ^]^ the challenge of achieving robust feature selection and capturing intricate microbe‐host phenotype relationships persists.^[^ ^34^, ^35^, ^36^ ^]^

In this study, a MIcrobial Cancer‐association Analysis using a Heterogeneous graph transformer (MICAH) to identify intratumoral microbial communities associated with cancer was proposed. By integrating complex relationships among microbes and between microbes and hosts, our method can provide testable hypotheses for elucidating the biological mechanisms underlying cancer‐microbiome relationships. Specifically, we constructed a heterogeneous graph to represent the intricate relationships between microbes and their hosts, where the phylogenetic and metabolic relationships among microbial species were included as knowledge edges in the initial representation. Based on this heterogeneous graph representation, we deployed a graph transformer to capture the links between microbes and multiple cancer types, enabling the interpretation of the relevance between microbial communities and host phenotypes. Finally, we identified cancer‐associated microbial communities for each cancer type by selecting communities of statistically significant species with high attention scores. We applied MICAH to a bacterial dataset of 5 cancer types in TCMA^[^ ^10^ ^]^ (Colon adenocarcinoma, abbr. COAD; Esophageal carcinoma, abbr. ESCA; Head and Neck squamous cell carcinoma, abbr. HNSC; Rectum adenocarcinoma, abbr. READ; and Stomach adenocarcinoma, abbr. STAD) and fungi datasets from 5 sequencing centers,^[^ ^1^ ^]^ and demonstrated its high reproducibility in identification of microbial communities associated with cancer. Compared to eight existing methods, MICAH achieved promising performance in identifying highly biologically meaningful cancer‐associated microbial communities.

Furthermore, we explored the effects of identified species from MICAH on host gene expression and found genes correlated with the abundance of microbial species were enriched for cancer‐associated cellular processes, including cell proliferation and invasion, inflammatory pathways, and immune system activation. We used mouse experiments and validated that *Blautia* species can enhance immunotherapeutic responses in a model of colorectal cancer. These findings suggested the high explainability of microbial communities identified by MICAH, which will contribute to the elucidation of host‐microbiome interactions associated with cancer. We believe the proposed graph neural network (GNN) framework is highly effective for identifying cancer‐associated microbial communities.

## 2 Results

### 2.1 MICAH is a GNN Framework for Identifying Cancer‐Associated Intratumoral Microbial Communities

**Figure 1:**
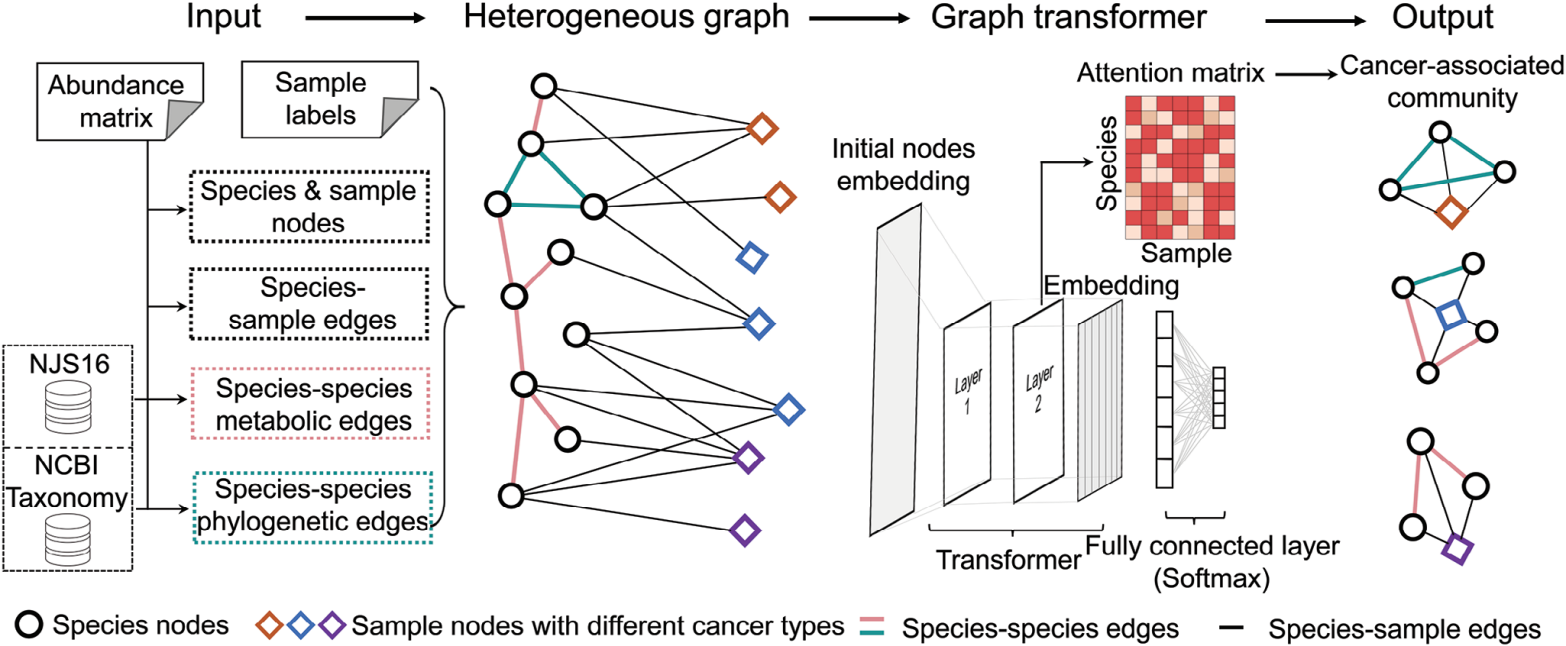
The MICAH framework. The required inputs are an abundance matrix and sample labels (i.e., cancer types of samples, represented in different colors such as orange, blue, and purple here). Then, we consider all species and samples included in the abundance matrix as species and sample nodes, respectively. The species‐species edges are constructed based on 2 existing databases, NJS16 (metabolic relationships as pink edges) and NCBI taxonomy database (phylogenetic relationships as green edges), respectively. The species‐sample edges (black edges) are constructed based on the abundance of each species within the sample. By this, we can obtain a heterogeneous graph with 2 types of nodes and 3 types of edges. Next, we use a two‐layer graph transformer to update node embeddings and a fully connected layer based on updated node embeddings for sample node classification. Finally, the attention scores of species to samples from the well‐trained model are extracted for the inference of microbial communities associated with different cancer types. The outputs are subsets of microbial species corresponding to different cancer types, i.e., the cancer‐associated microbial communities. For example, the cancer type orange is associated with a microbial community consisting of 3 species that have phylogenetic relationships.

MICAH takes known cancer labels for samples and a microbial abundance matrix as required input for the model training, as well as metabolic and phylogenetic relation matrices as optional input. The abundance matrix is organized with species as rows and samples as columns, where each element denotes the abundance of a particular microbial species within its corresponding sample. The metabolic/phylogenetic relation matrix indicates whether there is a metabolic/phylogenetic relationship between 2 species, with a value of one indicating a relationship and zero indicating no relationship. The output of MICAH is the microbial community of each cancer type, which can be prioritized as potential candidates associated with cancer occurrence, development, and treatment for future mechanistic studies. The overall framework consists of 4 steps (**Figure** **1**): i) Derive nodes and edges from the input abundance matrix; ii) Construct a microbial species‐cancer sample heterogeneous graph; iii) Train a graph transformer for cancer sample classification; and iv) Output the cancer‐associated microbial communities. First, MICAH detects microbial species and cancer samples within the input abundance matrix as species and sample nodes, respectively. The species‐sample edges are constructed based on the abundance of each species within the sample, with an edge created if the abundance is non‐zero. The species‐species edges are constructed based on the metabolic and phylogenetic relationships among species. If the user does not provide metabolic and phylogenetic relation matrices, MICAH will assess the relationships automatically using 2 existing databases, NJS16^[^ ^37^ ^]^ and NCBI taxonomy database.^[^ ^38^ ^]^ NJS16 contains 4400 metabolic interactions among 570 species while the NCBI taxonomy database contains the phylogenetic relationships among 25 434 species of bacteria and archaea. Both the metabolic and phylogenetic relationships assessed by MICAH were used in this study. Then, all nodes and edges form a heterogeneous graph, and a graph transformer is trained for sample classification. The attention is introduced to assess the contribution of species to samples. Finally, we identify the microbial communities that consist of species with significantly high attention scores for each cancer type as the cancer‐associated microbial communities. Details can be found in Experimental Section.

### 2.2 MICAH Consistently Outperformed other Methods in Terms of Reproducibility of Identified Cancer‐Associated Microbial Species

**Figure 2:**
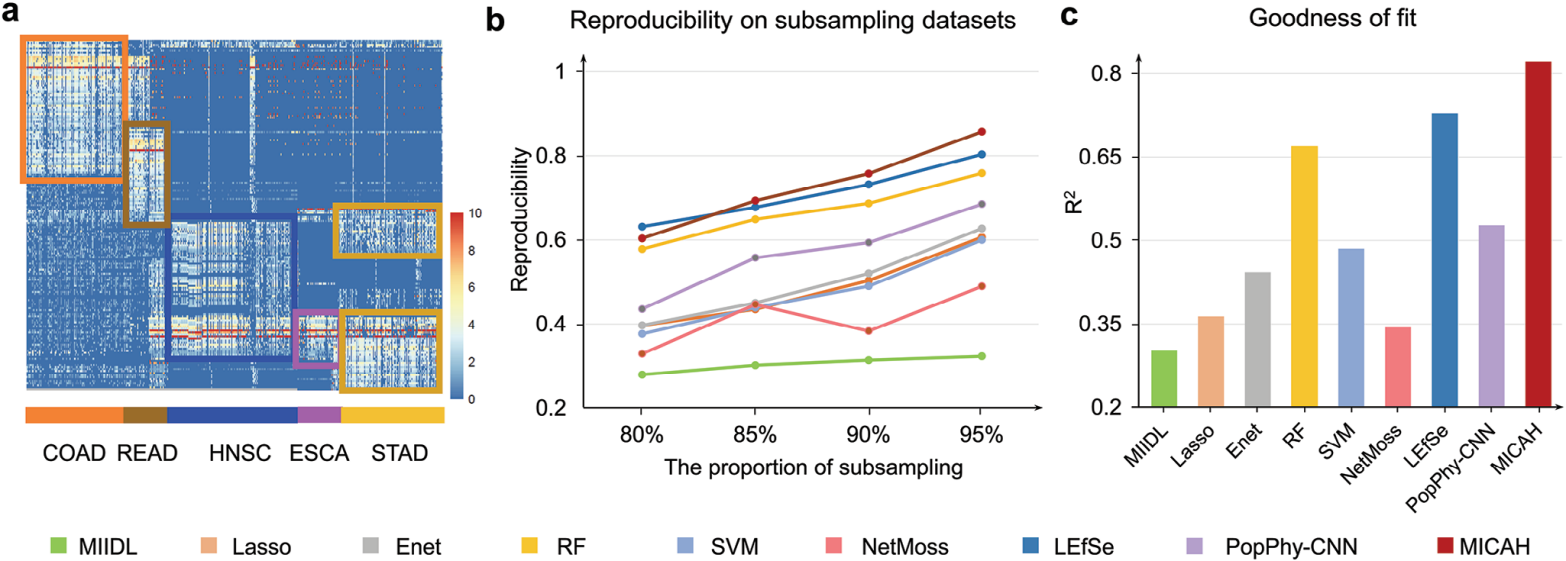
Results of classification and reproducibility performance. a) Attention score visualization. Each row is a species and each column is a sample. The elements are the normalized attention scores from each species to each sample. b) Reproducibility of MICAH and eight other competing methods in different subsampling datasets of TCMA. c) The goodness of fit with the line *y *= *x* of rank plots for the eight methods.

The cancer‐associated microbial species were identified based on the attention learned from MICAH. We applied MICAH to the bacterial dataset in TCMA and visualized the attention scores of microbial species to samples, by which distinct checkerboard structures of the 5 cancers were observed (**Figure** **2**a). This suggests our proposed framework can capture the discriminating characteristics of each cancer from the microbial data. It also indicates that the attention mechanism used in MICAH is highly interpretable for the relationships between microbial species and cancer.

We further measured the reproducibility of identified communities from MICAH. Ideally, the true characteristics of each cancer type should not be sensitive to sample variations. Data perturbation, such as changing or taking out a small number of samples, should not significantly alter the identified microbial characteristics. To test this, we first randomly selected a certain proportion of samples from the original TCMA dataset, forming a “new” dataset. This process was repeated 50 times and generated 50 datasets with a small fraction of differential samples. MICAH identified the intratumoral microbial characteristics, as well as eight other competing methods, i.e., Lasso, Enet, RF, SVM, MIIDL, PopPhy‐CNN, LEfSe (a representative statistics‐based method), and NetMoss (a representative network‐based method). A reproducibility index was then used to measure the similarity of identified characteristics across the 50 datasets (Experimental Section). Results indicated that MICAH has higher reproducibility than other methods for small sample variations (Figure 2b). Nevertheless, when the proportion of subsampling was less than 85%, the reproducibility of MICAH was slightly lower than the statistics‐based method, LEfSe. This may be due to the deep‐learning model's inherent limitations in sample size requirements. Further observation on the identified species found the concentration of the plots for the ranks of identified microbes from MICAH was highest ≈45° line (R^2^ = 0.8216), followed by LEfSe (R^2^ = 0.7289) and RF (R^2^ = 0.6689), while those of other methods seem spread with R^2^ < 0.55 (Figure 2c). This indicated that MICAH retained the ranks of the selected characteristics well. Additionally, we calculated the reproducibility of identified microbial species based on the 5 fungi datasets (Datasets in Experimental Section), respectively. We found that MICAH still suggested higher reproducibility compared to other methods. Details are shown in Table S1 (Supporting Information). Notably, LEfSe captured no cancer‐associated fungi. Observation of the fungi data characteristics found that this might be due to the high sparsity of the fungal datasets. Taken together, our findings demonstrate that MICAH is more reproducible in identifying cancer‐associated microbial characteristics than other existing methods.

### 2.3 The Microbial Communities Identified by MICAH have High Biological Relevance to 5 Cancer Types

**Figure 3:**
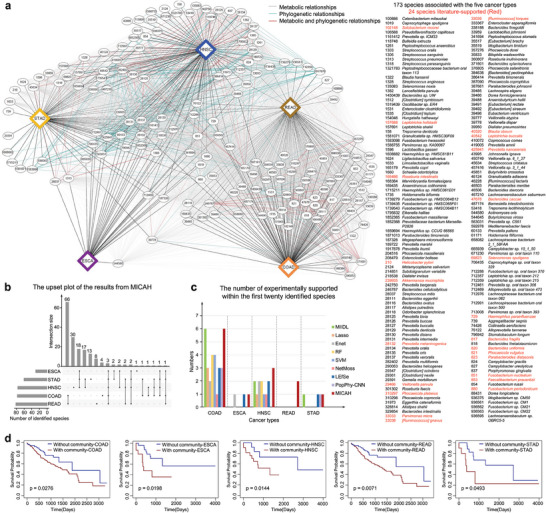
Identified microbial communities from MICAH. a) Microbial communities for the 5 cancer types. b) Distribution of the identified microbial communities across the different cancer types, shown in an upset plot of the 5 microbial communities. c) Comparison of literature‐supported species identified by MICAH and eight other competing algorithms. d) Kaplan‐Meier survival curves for the 5 identified microbial communities (the log‐rank test).

We observed the microbial communities from MICAH and explored their biological relevance to the 5 cancer types in TCMA. Overall, MICAH identified microbial communities that consist of 78, 30, 77, 82, and 61 microbial species for COAD, ESCA, HNSC, READ, and STAD, respectively (**Figure** **3**a; Table S2, Supporting Information). The COAD and READ‐associated communities shared more microbial species, while the other 3 were more similar (Figure 3b). Intuitively, this is reasonable since both COAD and READ begin in the large intestine. Moreover, a previous comprehensive molecular analysis has found that ESCA can be divided into 2 subtypes: one is similar to STAD and the other resembles some cancers such as HNSC.^[^ ^39^ ^]^ The current study did not distinguish between the subtypes of ESCA. Therefore, the characteristics of the 3 cancer types may appear similar. We further analyzed the reliability of identified microbial characteristics based on previous studies. We cross‐referenced the Peryton database^[^ ^40^ ^]^ for literature‐supported microbe‐disease associations with the intratumoral microbial species associated with the 5 cancer types. Of the 72, 2, 12, 4, and 2 species in COAD, ESCA, HNSC, READ, and STAD were included in the TCMA dataset, respectively, 64.72% were captured by MICAH successfully (Table S3, Supporting Information). Moreover, MICAH identified more literature‐supported species compared to the other eight methods (Figure 3c).

To examine whether identified communities from MICAH were associated with cancer prognosis, we analyzed the overall survival (OS) of the patients from TCGA Pan‐Cancer Clinical Data Resource.^[^ ^41^ ^]^ A log‐rank test was used to compare the survival distributions of patients with and without the identified communities (Experimental Section). Interestingly, the OS of patients in each pair of groups (with or without the identified microbial communities) was significantly different, with a log‐rank *p*‐value < 0.05 (Figure 3d). This result indicates that MICAH has the potential to contribute to the identification of prognostic characteristics of cancers.

### 2.4 Source Tracking Analysis Revealed that Cancer‐Associated Microbial Communities Were Primarily Attributed to the Microbial Communities Present on the Organ Affected by the Type of Cancer

**Figure 4:**
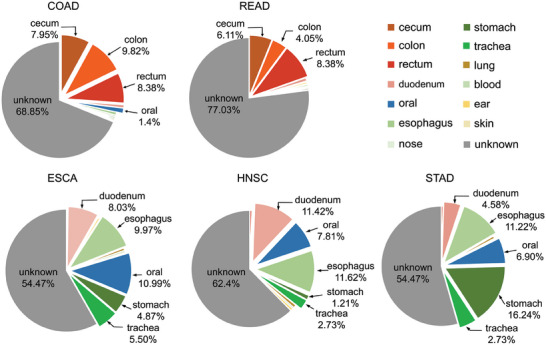
Body site attribution for samples from patients with the 5 cancers. Each pie chart shows the contribution of the microbial species at these sites to the corresponding cancer type.

To explore the formation of cancer‐associated microbial communities, we estimated the body sites with potential contributions.^[^ ^42^ ^]^ We collected microbial data from 13 human body sites related to health cohorts, including the cecum, colon, rectum, duodenum, blood, ear, esophagus, lung, nose, oral, skin, stomach, and trachea, from mBodyMap,^[^ ^43^ ^]^ and summarized the data at the species level. The species‐level data from the 13 body sites served as “sources,” while microbial data from cancer samples in TCMA were considered “sinks.” We used the SourceTracker2 tool with default parameter settings to evaluate the contribution of each source to each sink. The fractional contributions of each source to sinks with the same cancer are shown in **Figure** **4** and Table S4 (Supporting Information). Overall, the results revealed that the major known contributor to a certain cancer‐associated microbial community is those on the organs affected by the type of cancer. Specifically, the large intestine, including the cecum, colon, and rectum, was found to be the primary known contributor to COAD and READ. This suggests that microbial communities from the large intestine may have played a vital role in the development of these 2 types of cancer. Different from this, the major known contributors of ESCA and HNSC were found to be the oral cavity, esophagus, and duodenum. While the microbiome on the first 2 body sites has been reported to be associated with the development of ESCA and HNSC,^[^ ^44^, ^45^, ^46^ ^]^ the duodenum has not been previously identified as a significant contributor. Further investigation is needed to explore the underlying mechanisms of this association. For STAD, the stomach microbiome was found to be the most important contributor. This makes sense since *Helicobacter pylori*, a bacterium known to cause inflammation in the stomach, has been identified as a major factor in the development of STAD.^[^ ^47^ ^]^ Taken together, these results highlight the significance of intratumoral microbiomes in the occurrence and development of cancer. The identification of specific microbial communities associated with different types of cancer may provide insights into the underlying mechanisms of cancer development and lead to the development of novel prevention and treatment strategies.

### 2.5 The Attention Mechanism and Integration of Phylogenetic and Metabolic Relationships Benefit the Identification of Cancer‐Associated Microbial Communities in MICAH

**Figure 5:**
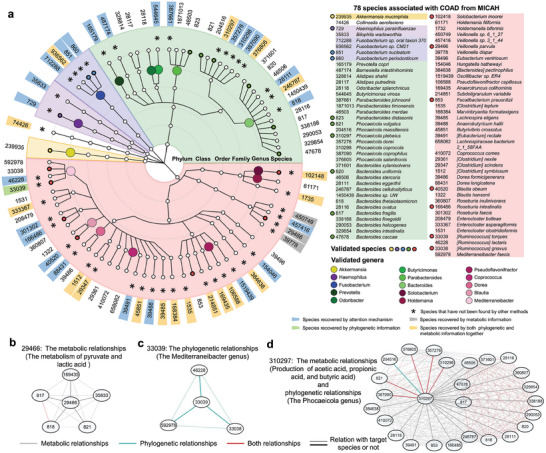
Identified communities of COAD. a) Phylogenetic tree of identified species. The rings from inner to outer indicate the taxonomic rank of the microbes, from phylum to species, respectively. The colored dots on the genus and species rank rings indicate the microbes that have been validated by previous studies, where large ones are validated at the genus level and small ones at the species level. An asterisk (*) is used to indicate the species uniquely found by MICAH. The external ring shows the annotation of each species using NCBI taxonomy ID. The colors on the IDs indicate why the species can be found by MICAH. b) Metabolic relationships of *Veillonella parvula*. c) Phylogenetic relationships of [*Ruminococcus*] *torques*. d) Metabolic and phylogenetic relationships of *Phocaeicola plebeius*.

The significant differences between MICAH and previous methods are that our framework integrates phylogenetic and metabolic relationships among species and uses attention scores to identify cancer‐associated microbial communities. We next explored whether these improvements contribute to reliable results in the widely studied cancer, COAD. We examined all 78 species in the COAD‐associated community from MICAH and compared them with those identified by the eight other methods. MICAH identified 46 unique species (**Figure** **5**a), of which nine have previously been demonstrated to be associated with COAD, and 17 species belong to genera that have also been shown to be associated with COAD. These findings indicate that MICAH can provide new and valuable insights into cancer‐microbiome studies.

To investigate why MICAH can find the species, we additionally identified COAD‐associated microbial species under 3 scenarios: i) using our framework but not integrating phylogenetic and metabolic relationships, referred to as MICAH without P‐M; ii) using our proposed framework without metabolic relationships, referred to as MICAH without *M*; and iii) using our proposed framework without phylogenetic relationships, referred to as MICAH without *P*. We first compared the species identified by MICAH without P‐M with those identified by the 8 other methods and MICAH. Results revealed that 24 species are uniquely identified by MICAH and MICAH without P‐M (Figure 5a). From this, we inferred that the 24 species primarily benefited from the attention mechanism without relying on any metabolic or phylogenetic relationships (Table S5, Supporting Information). This suggests that the attention mechanism contributes significantly to revealing the relationships between microbial species and cancer compared to the methods based solely on relative abundance. Then, we compared identified species from MICAH without P and MICAH without M to explore the role of metabolic and phylogenetic relationships. We found that 3 species (*Veillonella parvula, Veillonella dispar, and Veillonella sp. 6_1_27*) were identified by MICAH without *P* but could not be identified by MICAH without *M*, while one species ([*Ruminococcus*] *torques*) was identified by MICAH without *M* but not by MICAH without *P* (Figure 5a). From this, we speculate that the former 3 species tend to benefit from metabolic relationships, while the latter one species could benefit from phylogenetic relationships. We further analyzed the underlying metabolic relationships and phylogenetic relationships of these species and found that the speculation was reasonable.

For example, the species *Veillonella parvula* closely interacts with 5 other species, jointly involved in pyruvate and lactic acid metabolism (Figure 5b). Two of the 5 species (*Bacteroides fragilis* and *Phocaeicola vulgatus*) have been identified as associated with COAD by MICAH and eight competing methods. This implies the 2 species have distinctive characteristics in the COAD samples and can thus be captured by all methods. Given the heterogeneous graph representation and graph transformer used in MICAH, it was rational to infer that the metabolic relationship between *Veillonella parvula* and the 2 species may enhance less prominent features originally of *Veillonella parvula* associated with COAD, leading to its identification with COAD. Similarly, we showed the phylogenetic network of the species ([*Ruminococcus*] *torques*) that benefits from phylogenetic relationships (Figure 5c). This species belongs to the *Mediterraneibacter* genus, under which 2 species ([*Ruminococcus*] *gnavus* and *Mediterraneibacter faecis*) have been found by all methods. Therefore, [*Ruminococcus*] *torques* were identified by integrating with phylogenetic relationships.

Notably, not all microbial species with metabolic and phylogenetic relationships can be identified with the species of “distinctive characteristics.” The integration of metabolic and phylogenetic relationships can only help to enhance the COAD‐related characteristics by complementing the main attention mechanism, enabling the identification of species that would otherwise be difficult to find. Eighteen other species can only be found while integrating both phylogenetic and metabolic relationships (Table S5, Supporting Information). We believe they were contributed by integrating both phylogenetic and metabolic relationships. For example, *Phocaeicola plebeius* belongs to the Phocaeicola genus and is related to the production of many short‐chain fatty acids, such as acetic acid, propionic acid, and butyric acid (Figure 5d). These relations collectively led to the discovery of *Phocaeicola plebeius* as a COAD‐associated species. Overall, the integration of phylogenetic and metabolic relationships among species helps researchers identify microbial communities associated with cancer. It also makes the results of MICAH more explainable and understandable while investigating the biological mechanisms of cancer‐microbiome relationships.

### 2.6 The Microbial Communities Identified by MICAH were Associated with Host Gene Expression

**Figure 6:**
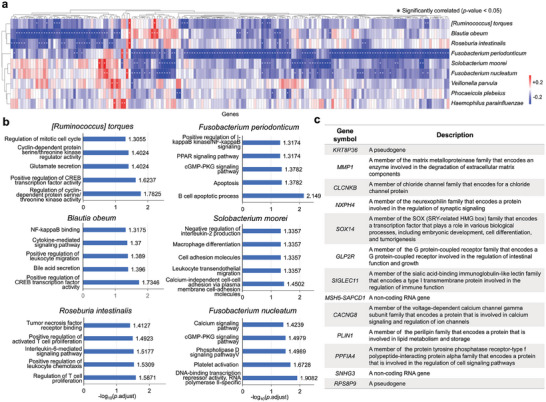
Association between microbial species from MICAH and host gene expression. a) Correlation between the abundance of identified species and the expression of DEGs for nine experimentally supported species uniquely found by MICAH. Each row is a species and each column is a gene. Cells are colored according to the Spearman correlation coefficient, in which an asterisk (*) indicates the correlation is statistically significant based on a two‐sided test based on a t‐distribution (with *p*‐value < 0.05). b) Enriched pathways of negatively correlated genes of 6 species. c) Positively correlated genes and their functions.

To explore whether microbial communities identified by MICAH have an impact on host gene expression and biological pathways in COAD, we examined the correlation between the abundance of identified species and the expression of differentially expressed genes (DEGs) between COAD tumors and normal samples.^[^ ^10^ ^]^ Results showed that the microbial species were significantly associated with specific DEGs (Figure S1, Supporting Information). Specifically, we focused on the nine literature‐supported species uniquely found by MICAH (**Figure** **6**a) and explored functions of genes significantly correlated with these species (with *p*‐value < 0.05, a two‐sided T‐test). Pathway analyses were first performed for the negatively correlated genes of each species. We displayed the first 5 pathways or biological processes of 6 species based on adjusted *p*‐values, while the other 3 did not show significant results (Figure 6b). Since the number of positively correlated genes of all species was limited, their functions were studied individually (Figure 6c). The results suggest that the significantly correlated genes tend to be involved in COAD‐associated cellular processes related to cell proliferation and invasion, inflammatory pathways, and immune system activation. We additionally focused on the species that were first found to be associated with COAD, and pathway analysis of the correlated genes came to similar conclusions (Figure S2, Supporting Information). This revealed an underlying mechanism that microbial species could be associated with cancer development and progression by modulating host gene expression.^[^ ^10^ ^]^ These findings provide insights into cancer prevention and treatment by targeting microbial communities or their effects on host gene expression. Nevertheless, the occurrence and progression of cancer is indeed a complex process that involves various factors. The relationships between microbes and host genes can either promote or inhibit cancer, relying on specific genes and pathways involved. For example, genes negatively related to the abundance of *Fusobacterium nucleatum (F. nucleatum)* were enriched for platelet activation and the cGMP‐PKG signaling pathway. Platelet activation can lead to the release of platelet‐derived growth factors and vascular endothelial growth factors, which can directly stimulate the growth of cancer cells and indirectly promote the development of new blood vessels to support tumor growth.^[^ ^48^ ^]^ In contrast, activation of the GMP‐PKG pathway inhibits cancer cell growth by regulating cell cycle progression and inducing apoptosis.^[^ ^49^ ^]^ The overall observation of 13 genes with positive correlations to nine species shows that ten of them exhibited a significant negative correlation with other species, indicating a distinct role of microbial species in COAD development. Therefore, a better understanding of the intricate interaction mechanisms underlying microbiome and cancer is crucial for designing effective strategies for cancer prevention and treatment.

### 2.7 Experimental Validation of the MICAH‐Identified Blautia‐Immune Relationship Demonstrates Blautia Supplementation Affects Response to Immunotherapy

We further investigated the associations between microbial species identified by MICAH and tumors. Our computational analyses revealed a potential impact of microbial species on host tumors through immune mechanisms. Consequently, we initiated mouse experiments on the functional impact of microbial species on tumors. Practical considerations, including the availability of experimental tools and expertise, prompted us to use *Blautia obeum (B. obeum)* and COAD as a starting point for our investigations. Specifically, we hypothesized that the relationship MICAH identified between *Blautia* and “positive regulation of leukocyte migration” could be observed by affecting immune infiltration in tumors containing *Blautia*. Therefore, treatments that rely on immune infiltration (e.g., immunotherapy) should be affected by *Blautia* colonization. Other microbial species identified by MICAH merit similar scrutiny and can be the subjects of future studies.

**Figure 7:**
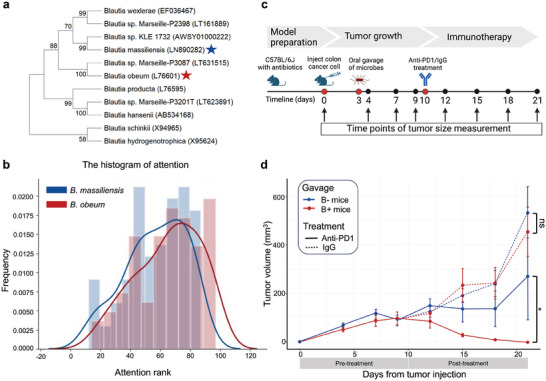
Investigation of microbial species from MICAH and COAD. a) Phylogenetic consensus tree of *Blautia* species included in TCMA based on 16S rRNA gene sequences. The tree was reconstructed with the neighbor‐joining algorithm. Bootstrap values calculated for 1000 subsets are shown at branch nodes. b) The distributions histogram of attention rank of *B. obeum* (red) and *B. massiliensis* (blue) in COAD samples. The attention rank in the horizontal axis represents the maximum attention rank selected from the multi‐head attention mechanism for each species to each COAD sample. c) Experimental schema comprising the model preparation phase, tumor growth phase, and immunotherapy phase. In the preparation phase, C57BL/6J mice (*n *= 22) received an antibiotic cocktail in their drinking water, alternating with regular water over 3 cycles. Subsequently, 1e6 mc38 cells were injected into mice on day 0. Oral gavage of stool slurry was administered with (*n *= 10) or without (*n *= 12) *B. massilliensis* strain on day 3. Mice were treated with anti‐PD1 antibody or isotype control (IgG) on day 10. Tumor sizes were measured every 3 days. d) Quantification of tumor volume (mm^3^) in mice from day 0 to day 21. Data are presented as mean ± standard error of the mean (SEM) shown as blue and red dots. Prior to treatment, there were 2 groups of mice (blue: B‐; red: B+). Post‐treatment, mice were categorized into 4 experimental conditions (blue solid line: B‐ with anti‐PD1; blue dashed line: B‐ with IgG; red solid line: B+ with anti‐PD1; red dashed line: B+ with IgG). Post‐treatment mice were analyzed using a longitudinal mixed‐effect model with Sidak's correction for multiple comparisons. *p < 0.05; ns, not significant. B‐ mice: *B. massillience* negative; B+ mice: *B. massillience* positive.

We employed a colonization approach by orally gavaging microbes to C57BL/6 mice with syngenetic, subcutaneous colorectal cancer (Experimental Section). Unfortunately, our attempts to obtain a strain of *B. obeum* were unsuccessful. Therefore, we opted for the closest available relative, *Blautia massilliensis (B. massiliensis)*, as depicted in **Figure** **7**a. This *Blautia* species is the most closely related taxon in The Broad Institute‐Open Biome Microbiome Library “diversity box,”^[^ ^50^ ^]^ and was described by Durand et al as a close relative of obeum.^[^ ^51^ ^]^ Given the focus on assessing species' contributions to samples, we additionally examined the distribution of attention score ranks for all species within the *Blautia* genus in COAD samples, which can be directed toward assessing the contribution of species to samples (Experimental Section). Our analysis indicated that the attention rank distributions of *B. obeum* and *B. massiliensis* are more similar to each other (Wasserstein distance of 7.0568, Figure 7b) than those of *B. obeum* and other species (Wasserstein distance > 8.034, Figure S3, Supporting Information). This suggested a closer relationship between *B. obeum* and *B. massiliensis* from a computational perspective.

Several studies have demonstrated that oral gavage of microbes can lead to tumor infiltration. For example, Bender et al. showed that a gavage with *Lactobacillus reuteri* led to tumor colonization that affected immunotherapy response only when the tumor microbes were present.^[^ ^52^ ^]^ Similarly, Vincent et al. demonstrated that a gavage with an engineered E coli led to tumor colonization that could enhance the tumor‐directed targeting of chimeric antigen receptor T cells.^[^ ^53^ ^]^ Thus, we used a synthetic colorectal cancer mouse model that is orally administered stool slurry either with (B+) or without (B‐) supplementation of *B. massilliensis*. Ten days post‐tumor induction, both B+ and B‐ mice were randomly subdivided to receive anti‐PD1 antibody or isotype control (Figure 7c). We collected the tumor tissues of 6 mice from both B+ and B‐ mice and quantified the microbial reads (Experimental Section). We observed *Blautia* RNA in the B+ mice versus its absence in the B‐ mice (Figure S4, Supporting Information), indicating the *Blautia* infiltration in the tumor tissues. Our results indicate that, prior to the initiation of immunotherapy, tumor volumes are significantly increased over time across all mice (*p* = 1.2e–15) (Figure 7d). No significant differences are observed between B+ and B‐ mice (*p* = 0.629, Table S6, Supporting Information), indicating that *B. massilliensis* does not directly influence tumor growth. However, a divergence in tumor volume between B+ and B‐ mice appears after giving the anti‐PD1 treatment on Day 10. A longitudinal mixed‐effects model was built to estimate the effects of experimental conditions (i.e., B+ mice with anti‐PD1, B‐ with anti‐PD1, B+ without treatment, B‐ without treatment), time, and their interactions on tumor volume (Table S7, Supporting Information). Notably, B+ mice receiving anti‐PD1 therapy exhibited a substantial reduction in tumor size (mean decrease of 270.17 mm^3^, *p* = 0.0175) compared to B‐ mice treated with anti‐PD1 (Figure 7d). This effect was not observed in untreated mice (*p* = 0.6488), suggesting that *B. massilliensis* alone does not mitigate tumor growth. Further ANOVA analysis also confirmed a significant association between the condition‐time interaction effect and tumor volume (*p* = 0.006, Table S8, Supporting Information). These findings suggest that while the presence of *B. massilliensis* in tumors does not directly inhibit progression, its supplementation could potentiate the efficacy of anti‐PD1 therapy. Consequently, *B. massilliensis* has the potential to enhance immunotherapeutic responses. While we acknowledge the limitations introduced by not using the exact strain observed in the tumor samples (e.g., via cultivation from tumor tissue), we used the closest available isolate. Being in the same genus as *B*. *obeum* increases the likelihood of acting via the same mechanism. We propose that future research efforts could involve obtaining specific strains of *B. obeum* for further validation, thereby enhancing the robustness of our findings.

## 3 Discussion

Although the cancer‐associated microbiome has been extensively studied for decades, the relationships between intratumoral microbes and cancer are far from fully understood.^[^ ^54^ ^]^ While existing tools have demonstrated considerable success in host phenotype classification, the challenge of achieving robust feature selection and capturing intricate microbe‐host phenotype relationships persists. To this end, we developed the MICAH framework to identify cancer‐associated intratumoral microbial communities. MICAH has 3 key features: i) an explainable deep learning framework to infer cancer‐associated intratumoral microbial communities; ii) the ability to abstract metabolic and phylogenetic relationships among all species in a graph representation learning framework to systematically reflect the intricate interactions within microbial communities; and iii) the introduction of the attention mechanism for holistic interpretation of the relevance between microbial species and sample phenotypes. We have successfully identified intratumoral microbial characteristics of 5 cancer types using MICAH. The success partially comes from the power of GNN for modeling complex relationships among samples, microbes, and cancer types, including their local and global dependencies. GNN is robust to noisy or incomplete data due to its ability to incorporate information from neighboring nodes in the graph. In addition, the heterogeneous graph transformer framework of GNN is computationally efficient and scalable to large graphs. It took about an hour to get the final results on the TCMA dataset. We believe that MICAH can contribute to understanding complex microbial communities, guide biologists in understanding cancer pathogenesis and provide potential microbial targets for cancer treatment. Furthermore, our framework can be extended to identify microbial signatures associated with different diseases and environmental exposure.

The MICAH results are notable for raising microbe‐gene interactions established through careful experimentation, as well as highlighting novel associations for future study. As an example of the former, MICAH observed strong relationships in COAD with *F. nucleatum*, which has a long history in COAD tumors. *F. nucleatum* binds to carbohydrates on the surfaces of tumors via Fap2 lectin^[^ ^55^ ^]^ and contributes to diverse phenotypic alterations, including a transition to a more mesenchymal‐like state.^[^ ^56^ ^]^ *F. nucleatum* was also found to affect the host therapeutic response in a mouse model. Infected CRC cells with Fn displayed resistance in response to Oxaliplatin and 5‐FU via autophagy activation.^[^ ^57^ ^]^ Notably, MICAH identified 2 oral‐pathogen members of the genus (*F. nucleatum* and *F. periodonticum*) whose interactions with tumor cells were distinct but shared enrichment of the cGMP‐PKG signaling pathway.

On the other hand, *Roseburia gnavus (R. gnanus)* is a gut commensal that does not, to our knowledge, have an established mechanism in the tumor microbiome. However, *R. gnavus* produces a pro‐inflammatory polysaccharide, glucorhamnan, that is recognized by innate immune cells via toll‐like receptor 4 (TLR4).^[^ ^58^ ^]^ MICAH revealed that tumors containing *R. gnanus* showed enrichment of genes that are required for TLR‐mediated signaling, the cyclin‐dependent protein kinases pathway.^[^ ^59^ ^]^ Furthermore, guided by the finding by MICAH, we experimentally validated the role of *Blautia* in modulating the response to anti‐PD1 therapy in colorectal cancer. These suggested that MICAH's attention mechanism of integrating microbe and gene information yields strong associations with plausible mechanisms for experimental follow‐up.

Novel to the tumor microbiome is the observation of *Roseburia intestinalis (R. intestinalis)*, a common gut commensal. *R. intestinalis* has been associated with many indicators of health, including improved response to treatment with immune‐checkpoint inhibitors,^[^ ^60^ ^]^ likely through butyrate‐mediated colonocyte activation.^[^ ^61^ ^]^ Here, we see that tumors containing *R. intestinalis* were enriched for genes in T‐cell activation pathways. This suggests that this microbe could be a target for rational manipulation to improve treatment outcomes.

However, this study has some limitations. First, MICAH relies on an existing database to assess metabolic relationships between species, which may not fully reflect the true metabolic activities within the community in specific cancers. Admittedly, it is a way to assess metabolic relationships to compensate for the lack of metabolic data. This issue can be addressed if the user can provide metabolic data for species‐species relationships assessment. Furthermore, the metabolic and phylogenetic relationships among species in this study are only a simplified representation, and their directionality and strength have not been considered. More orthogonal datasets that capture other types of interactions with the tumors should be considered, such as di‐nucleotide signaling, iron‐capture, oxygen‐tension effects, and co‐factor production. Moving forward, improving the representation and application of these relationships will be a key focus in achieving a more comprehensive understanding of how species interact and influence each other within microbial communities. Second, mechanistic interpretation of identified microbial communities to cancer remains difficult based only on the microbial data from bulk tissue sequencing. Spatial sequencing and single‐cell sequencing data provide a more comprehensive understanding of the spatial distribution of microbes within human tissue and their interactions with specific cell types.^[^ ^62^, ^63^ ^]^ By leveraging these data, researchers can gain insights into the microbial community structure, functions, and their relationships with host cells, leading to a deeper understanding of the role of the intratumoral microbiome in cancer development and progression. Third, we focus on cancer‐associated microbial species communities, while it is insufficient to understand microbial communities in species‐level resolution.^[^ ^64^ ^]^ Considerable genomic and phenotypic variability has been observed within species,^[^ ^64^ ^]^ and several species have both pathogenic and commensal strains.^[^ ^65^ ^]^ Therefore, a more detailed characterization of microbial communities for within‐species variation is necessary for cancer‐associated microbial signature exploration. Additionally, future studies should introduce time‐series data to explore microbial communities’ succession. This will improve our understanding of why microbes are present within the tumor and how they colonize there over time. Except for that, diverse and high‐quality data, such as longitudinally collected tissue microbiomes and paired clinical metadata (e.g., histological phases, serum biomarkers, and other clinical evaluation scores), are needed. We hope to integrate these data and modify our model to further elucidate the microbiome's role in cancer, predict microbial biomarkers highly associated with tumor regression or metastasis, and advance microbiome‐based approaches in oncology. A relative temporal encoding strategy can be used to model temporal dependencies in a GNN model, thereby capturing microbial longitudinal dysbiosis in cancer and improving the understanding of cancer development.

## 4 Experimental Section

### Datasets

Intratumoral microbial profile data across 5 cancer types with high bacterial loads, including COAD, ESCA, HNSC, READ, and STAD were utilized. The data were sourced from the TCMA database, previously published by Dohlman et al.^[^ ^10^ ^]^ Briefly, the intratumoral microbial data were originally isolated from whole genome sequencing data of tissue samples available in the TCGA. To ensure data quality, the authors of the TCMA database implemented a rigorous preprocessing pipeline, which involved the removal of host and contaminant information, as well as data normalization. These were achieved through sequencing alignments and their in‐house statistical model via comparative analyses of species prevalence in tissue and blood.^[^ ^10^ ^]^ The decontaminant step also mitigated potential batch effects arising from samples being sequenced at different centers.^[^ ^10^ ^]^ Through these efforts, the TCMA database has been a valuable public resource of decontaminated microbial profiles covering various taxonomic resolutions, ranging from phylum to species level.^[^ ^10^ ^]^ While the authors of TCMA also applied their model to other tumors, they found that other tumors had low microbial loads, yielding few statistically significant intratumoral microbial populations. This made it challenging to distinguish tissue‐resident species from contaminants. Therefore, they only published intratumoral microbial profiles for the 5 cancer types with high bacterial loads. For the investigation, the highest resolution available was specifically retrieved, i.e., species‐level intratumoral microbial abundance data from primary tumor tissues (Table S9, Supporting Information).^[^ ^10^ ^]^ A comprehensive species‐sample abundance matrix, encompassing a total of 1218 distinct microbial species and 512 samples was then compiled. Intratumoral fungi data from The Pan‐Cancer Mycobiome Atlas,^[^ ^1^ ^]^ and extracted fungal metagenomes profiling at the species level were additionally retrieved. By this, fungal abundance data of 2001 samples from primary tumor tissues, covering 24 cancer types was obtained. To mitigate batch effects, the samples were divided into 5 batches according to the source sequencing centers. Finally, 5 fungal species‐sample abundance datasets, named batch 1, batch 2, batch 3, batch 4, and batch 5, respectively were obtained.

### MICAH: A Framework to Identify Cancer‐Associated Intratumoral Microbial Communities

MICAH is an end‐to‐end GNN framework for cancer‐associated microbial community inference. This framework leverages a heterogeneous graph transformer (HGT) model to classify samples and employs attention scores obtained from the model to evaluate the relevance between microbial species and cancer types. The required input data of MICAH includes a vector *Y* representing sample cancer types and a microbial abundance matrix. The output was microbial communities composed of species with significantly high attention scores for each cancer type.

### Process the Input Data

For the input abundance matrix, a pre‐processing first was conducted: the rows that contain less than 0.05% non‐zero values are removed. Then, the matrix and obtained a new relative abundance matrix was re‐normalized, *A*_*M* × *N*_, where each row represents a microbial species, each column represents a sample, and the entry represents the relative abundance of a species in the corresponding sample. The abundance matrix used later refers to this preprocessed relative abundance matrix unless exceptions were mentioned.

Metabolic relationships between species were next assessed. Since microbial metabolomics data of most samples were not available,^[^ ^66^ ^]^ An existing database NJS16,^[^ ^37^ ^]^ which includes more than 4400 known metabolic interactions between 570 well‐studied microbial species, to assess metabolic relationships among species was relied on. The NJS16 database collected the experimentally verified metabolic relationships from the literature and listed the compounds produced or consumed by each microbial species. Metabolic relationships between species based on their associated compounds were estimated.^[^ ^67^ ^]^ If 2 species consumed the same compound, there was a metabolic competition between the 2 species;^[^ ^67^ ^]^ if a compound that one species needed to consume was exactly the one produced by the other species, there was a metabolic complementary relationship between the 2 species.^[^ ^67^ ^]^ Metabolic complementary or competition relationships here were not distinguished. In this way, a metabolic relation matrix could be obtained, $D_{M\times M}^{1}$, to represent metabolic relationships between species, where $d_{i_{1}i_{2}}^{1}=\;1$ if there is a metabolic relationships between the *i*_1_^*th*^ species and the *i*_2_^*th*^ species (*i*_1_ ≠ *i*_2_); otherwise, $d_{i_{1}i_{2}}^{1}=\;0$.

Phylogenetic relationships between species were measured using the NCBI taxonomy database. This database includes a hierarchical system of classification that was based on the principles of phylogenetic relationships, with each level representing a different degree of relatedness. The phylogenetic relationships that 2 species are in the same genus were considered^[^ ^38^ ^]^ (Section S1, Supporting Information). In this way, a phylogenetic relation matrix, $D_{M\times M}^{2}$, can be obtained, where $d_{i_{1}i_{2}}^{2}=\;1$ if the *i*_1_^*th*^ species and the *i*_2_^*th*^ species (*i*_1_ ≠ *i*_2_) are in the same genus; otherwise, $d_{i_{1}i_{2}}^{2}=\;0$.

For the label vector *Y*  = {(*y*_1_,…, *y_N_*)|*y_i_* ∈ {1, …, *C*}} , that indicates cancer types of *N* samples, one‐hot encoding to transfer it into a binary matrix was used ${\bar{Y}}^{N\times C}=\left\{{\left[y_{ic}\right]}_{N\times C}|y_{ic}\in \left[0,1\right],\;\forall i=1,\text{…},N,\;c=1,\text{…},C\right\}$, in which *C* is the number of cancer types of all samples. If the *i^th^* sample belonged to the *c^th^* cancer type, *y_ic_* =  1; otherwise *y_ic_* =  0.

### Construct a Microbial Species‐Sample Heterogeneous Graph

A microbial species‐sample heterogeneous graph *G* = (*V*, *E*, *P*, *Q*), where *V* is the node‐set, *E* is the edge set, and *P* and *Q* represent node type and edge type respectively was constructed.^[^ ^68^ ^]^ The heterogeneous graph *G* was composed of 2 types of node (microbial species nodes and sample nodes) and 3 types of edge (species‐sample edges and species‐species metabolic and phylogenetic edges), which were generated from the abundance matrix *A*_*M* × *N*_ and the obtained relation matrices, $D_{M\times M}^{1}$ and $D_{M\times M}^{2}$. Mathematically, $V=V^{s}\;\cup V^{p}$, where $V^{s}=\left\{v_{i}^{s}|i=1,2,\text{…},M\right\}$ denotes all microbial species nodes, and $V^{p}=\left\{v_{j}^{p}|j=1,2,\text{…},N\right\}$ denotes all sample nodes. $E=E_{1}^{ss}\;\cup E_{2}^{ss}\cup E^{sp}$, where $E_{1}^{ss}=\left\{e_{i_{1}i_{2}}^{ss}|\;i_{1},i_{2}\in \left\{1,2,\text{…},M\right\},\;and\;d_{i_{1}i_{2}}^{1}=1\;in\;D_{M\times M}^{1}\right\}$ and $e_{i_{1}i_{2}}^{ss}$ represents metabolic edge between specie nodes $v_{i_{1}}^{s}\in V^{s}$ and $v_{i_{2}}^{s}\in V^{s}$; $E_{2}^{ss}=\left\{e_{i_{1}i_{2}}^{\tilde{ss}}|\;i_{1},i_{2}\in \left\{1,2,\text{…},M\right\},\;\mathrm{and}\;d_{i_{1}i_{2}}^{2}=1\;in\;D_{M\times M}^{2}\right\}$, and $e_{i_{1}i_{2}}^{\tilde{ss}}$ represents phylogenetic edge unions between species nodes $v_{i_{1}}^{s}\in V^{s}$ and $v_{i_{2}}^{s}\in V^{s}$; $E^{sp}=\left\{e_{ij}^{sp}\left|\;i\in \left\{1,2,\text{…},M\right\},\;j\in \left\{1,2,\text{…},N\right\},\mathrm{and}\;a_{ij}\right\rangle 0\;in\;A_{M\times N}\right\}$ represents the edge between a species node $v_{i}^{s}$ and a sample node $v_{j}^{p}$.

### Train a Heterogeneous Graph Transformer for Cancer Sample Classification

A supervised GNN model and introduce node‐ and edge‐type dependent attention mechanisms to assess the relevance between microbial species and samples was proposed.

Considering the abundance matrix *A*_*M* × *N*_ is extremely sparse and noisy,^[^ ^69^ ^]^ 2 autoencoders to extract representative information as the initial embeddings of species and sample nodes in *G*, respectively were first used. The species autoencoder learns low‐dimensional features as species nodes embedding, which consists of an encoder with the transpose of *A*_*M* × *N*_, denoted as *A*_*M* × *N*_^*T*^, as the input layer, a hidden layer, and a decoder to restore the features in the hidden layer. The output layer is a reconstructed matrix $\bar{{A_{M\times N}}^{T}}$ of the same dimension with *A*_*M* × *N*_^*T*^. The mean square error between *A*_*M* × *N*_^*T*^ and $\bar{{A_{M\times N}}^{T}}$ is used as the loss function of the species autoencoder. Similarly, the sample autoencoder learns low‐dimensional features as sample nodes embedding, whose structure is the same as the species autoencoder, but the input is the matrix *A*_*M* × *N*_, and the output is $\bar{A_{M\times N}}$ of the same dimension with *A*_*M* × *N*_. The loss function is the mean square error between *A*_*M* × *N*_ and $\bar{A_{M\times N}}$. After such processing, a feature vector to each species and each sample node as the initial embedding was assigned.

A GNN model is applied to classify sample cancer types. *L* hidden layers are used to transmit and update the node features, and the default value of *L* is 2 in this work. A fully connected layer with *C* neurons is then connected, which uses the softmax activation function to predict the probability distribution over the *C* cancer classes. The final output is a matrix *P* ^*N* × *C*^ = {(*p*_1_, …, *p_N_*)^*T*^|*p_ic_* ∈ [0, 1],  ∀*i* = 1, …, *n*,  *j* = 1, …, *C*} , which can be used to predict sample labels. To alleviate the issue of data imbalance, Focal Loss as the classification loss function was used. Given the graph heterogeneity, the node‐ and edge‐type dependent attention mechanism is specifically introduced for node features update in the model.^[^ ^68^ ^]^ The next focus was on how to use attention mechanisms, do message passing and aggregation, and design loss functions.

Before the details, several definitions and symbols were given: 1) the mapping function of node and edge types. τ(*v*): *V* → *P* and ϕ(*e*): *E* → *Q* was defined as the mapping function of node types and edge types, respectively. 2) Source nodes and target nodes. When the focus was on a node, the node could be considered a target node, denoted as *v_t_*. If there was an edge between *v*_*s*_ and *v_t_*, the node *v* *_s_* could be considered as a source node of node *v_t_*. 3) Meta relation. It described a connection between a source node *v* *_s_* and a target node *v_t_*, using the node types of *v* *_s_* and *v_t_*, and edge types *e_st_* to represent, denoted as 〈τ(*v_s_*),ϕ(*e_st_*),τ(*v_t_*)〉.

### Step 1 Introducing Node‐ and Edge‐ Type Dependent Multi‐Head Attention Mechanism

Attention indicates the importance of a source node *v* *_s_* a target node *v_t_*. Multi‐head attention was a combination of multiple independent attention and helped to attend to parts of the feature differently.^[^ ^70^ ^]^ *h* attention heads were used and set *h* = 8 as default according to the grid search results.

When the attention value of a source node was calculated *v_s_* to a target node *v_t_*, the attention of each head is an independent output and then concatenated as an attention vector between verses and *v_t_* (Figure S5, Supporting Information). Specifically, the updated embedding of a target node *v_t_* and a source node *v* *_s_* the *l^th^* layer as $H^{l}\left[v_{t}\right]$ and $H^{l}\left[v_{s}\right]$ (*l*  =  0,  1,  … , *L*), respectively were denoted, where $H^{0}$ represents the initial node embedding. *d* is the dimension of $H^{l}\left[v_{t}\right]$. For the *k^th^* attention head in the *l^th^* HGT layer, $ATT\_head_{k}^{l}\left(v_{s},e_{st},v_{t}\right)$(*k*  =  1, 2, … , *h*), node‐type‐dependent linear projection functions ($K\_linear_{\tau (v_{s})}^{k}$ and $Q\_linear_{\tau (v_{t})}^{k}$) to map the embedding of the source node and target node in the *k^th^* head and (*l* − 1)^*th*^ layer, respectively were used, obtaining the *k^th^* Key vector *K^k^* (*v_s_*) and Query vector *Q^k^* (*v_t_*).

(1) $${K_{linear}}_{\tau \left(v_{s}\right)}^{k}:\;R^{d}\to R^{\frac{d}{h}}$$

(2) $${Q_{linear}}_{\tau \left(v_{t}\right)}^{k}:\;R^{d}\to R^{\frac{d}{h}}$$

(3) $$Q^{i}\;\left(v_{t}\right)={Q_{linear}}_{\tau \left(v_{t}\right)}^{k}\;\left(H_{k}^{\left(l-1\right)}\left[v_{t}\right]\right)$$

(4) $$K^{k}\;\left(v_{s}\right)={K_{linear}}_{\tau \left(v_{s}\right)}^{k}\;\left(H_{k}^{\left(l-1\right)}\left[v_{s}\right]\right)$$

Then, the similarity between the Key vector and Query vector is calculated as $AT{T_{head}}_{k}^{l}\left(v_{s},e_{st},v_{t}\right)$. Given the different edge types between the source node and the target node, an edge‐type‐dependent matrix $W_{\phi (e_{st})}^{ATT}\in R^{\frac{d}{h}\times \frac{d}{h}}$ is applied here to capture distinct relationships even between the same node‐type pair.

(5) $$\begin{array}{c} AT{T_{head}}_{k}^{l}\left(v_{s},e_{st},v_{t}\right)=\left(K^{k}\left(v_{s}\right)W_{\phi \left(e_{st}\right)}^{ATT}Q^{k}{\left(v_{t}\right)}^{T}\right)\;\cdot \frac{\mu \langle \tau \left(v_{s}\right),\phi \left(e_{st}\right),\tau \left(v_{t}\right)\rangle }{\sqrt{d}} \end{array}$$

where μ〈τ(*v_s_*),ϕ(*e_st_*),τ(*v_t_*)〉 is a prior tensor to denote the significance of each meta‐relation 〈τ(*v_s_*),ϕ(*e_st_*),τ(*v_t_*)〉, serving as an adaptive scaling to the attention.^[^ ^68^ ^]^ Next, the multiple attention heads are concatenated and an attention vector for each node pair is obtained, denoted as $\underset{k}{∥}\left(AT{T_{head}}_{k}^{l}\left(v_{s},e_{st},v_{t}\right)\right)$, where $\underset{k}{∥}$ is a concatenation function. Finally, all attention was gathered from vectors from all source nodes of a target node *v_t_* and used softmax, as Formula (6). In this way, $\sum_{v_{s}\in N\left(v_{t}\right)}ATT^{l}\left(v_{s},e_{st},v_{t}\right)$ is equal to 1, which facilitates the measurement of the importance of a source node *v* *_s_* a target node *v_t_*.

(6) $$ATT^{l}\;\left(v_{s},e_{st},v_{t}\right)=\underset{v_{s}\in N\left(v_{t}\right)}{\mathrm{Softmax}}\left(\underset{k}{∥}\left(AT{T_{head}}_{k}^{l}\left(v_{s},e_{st},v_{t}\right)\right)\right)$$

where $N(v_{t})$ is a set of source nodes of *v_t_*.

### Step 2 Message Passing and Aggregation

In this step, the message passed from a source node *v_s_* to a target node *v_t_* was first extracted. Then, the message was aggregated from all source nodes of *v_t_* using the corresponding attention as weight, obtaining updated embedding for the target node *v_t_*.

Specifically, for the *k^th^* message head in the *l^th^* HGT layer from *v* *_s_* to *v_t_*, $MS{G_{head}}_{k}^{l}$(*k* = 1, 2, … , *h*), a node‐type‐dependent linear projection function, ${V_{linear}}_{\tau (v_{s})}^{k}$, to map the embedding in the *k^th^* head and (*l* − 1)^*th*^ layer, obtaining the *k^th^* Value vector *V^k^* (*v_s_*) was useed.

(7) $${V_{linear}}_{\tau \left(v_{s}\right)}^{k}:\;R^{d}\to R^{\frac{d}{h}}$$

(8) $$V^{k}\;\left(v_{s}\right)={V_{linear}}_{\tau \left(v_{s}\right)}^{k}\;\left(H_{k}^{\left(l-1\right)}\left[v_{s}\right]\right)$$

Incorporating an edge‐type‐dependent matrix $W_{\phi (e_{st})}^{MSE}\in R^{\frac{d}{h}\times \frac{d}{h}}$, the Formula (9) was used to compute $MS{G_{head}}_{k}^{l}\left(v_{s},e_{st},v_{t}\right)$. All *h* message heads are concatenated for the overall message of the node pair *v* *_s_* and *v_t_*, *MSG^l^* (*v_s_*,*e_st_*,*v_t_*).

(9) $$MS{G_{head}}_{k}^{l}\;\left(v_{s},e_{st},v_{t}\right)=V^{k}\;\left(v_{s}\right)W_{\phi \left(e_{st}\right)}^{MSE}$$

(10) $$MSG^{l}\;\left(v_{s},e_{st},v_{t}\right)=\underset{k}{∥}\left(MS{G_{head}}_{k}^{l}\left(v_{s},e_{st},v_{t}\right)\right)$$

Finally, for a specific target node *v_t_*, the message of all of its source nodes was aggregated for embedding update (Formula (12)). The attention (after the softmax function) was used as the weight of the passing message from the corresponding source node, θ is a trainable parameter, and *ReLU* is the activation function.

(11) $$\tilde{H^{l}}\;\left[v_{t}\right]=\underset{v_{s}\in N\left(v_{t}\right)}{Aggregate}\;\left(ATT^{l}\left(v_{s},e_{st},v_{t}\right)\cdot MSG^{l}\left(v_{s},e_{st},v_{t}\right)\right)$$

(12) $$H^{l}\;\left[v_{t}\right]=\theta \left(ReLU\left(\tilde{H^{l}}\left[v_{t}\right]\right)\right)+\left(\theta -1\right)H^{l-1}\left[v_{t}\right]$$

After stacking information via all *L* layers, the final node embeddings are used for downstream sample classification.

### Step 3 Designing the Loss Function

The model is to solve a sample classification problem, and each sample has a known cancer label. Data imbalance is a common occurrence, which makes it challenging to accurately predict labels. To address this issue, Focal Loss to quantify the differences between the predicted labels and true labels (Formula (13)) was used.

(13) $$Loss_{focal}=-\frac{1}{N}\sum_{i\;=\;1}^{N}\sum_{c\;=\;1}^{C}\left[\alpha_{c}\times {\left(1-p_{ic}\right)}^{\gamma }\times y_{ic}\times \log\left(p_{ic}\right)\right]$$

(14) $$\alpha_{c}=1-\frac{\sum_{i\;=\;1}^{N}y_{ic}}{N}$$

where *N* is the total number of samples; α_*c*_ represents the weight of the *c^th^* cancer type used to balance the sample number of each class; $y_{ic}\in {\bar{Y}}^{C\times N}$; *p_ic_* ∈ *P* ^*C* × *N*^; γ is a tunable parameter (γ ≥ 0), and a higher value makes the model focus more on hard‐to‐classified samples Intuitively, this function assigns more weights to hard or easily misclassified samples than well‐classified samples.

To mitigate overfitting while training the model and make the model capture more comprehensive distinguishable characteristics of each cancer type, a regularizer was added such that the final embeddings of all nodes can reflect the initial feature as much as possible, where *KL*(*,*) represents the Kullback‐Leibler divergence; *A*_*M* × *N*_ is the preprocessed abundance matrix; *S_encoder_* and *P_encoder_* represent the final embedding matrices of species and samples, respectively.

(15) $$Loss_{KL}=KL\left(S_{encoder}{P_{encoder}}^{T},\;A_{M\times N}\right)$$

The overall loss function, denoted as *Loss*, is obtained via integrating the classification loss term and the regularization term, in which α is the regularization factor and the default value is 0.003 considering the balance of scale between the classification loss term and the regularization term.

(16) $$Loss\;=Loss_{focal}\;+\alpha \times Loss_{KL}$$

### Output the Cancer‐Associated Microbial Communities

In the HGT model, the attention score is considered a form of quantitative value to assess the importance of a source node to a target node. The multi‐head attention of each species node was extracted $v_{i}^{s}\in V^{s}$ to each sample node $v_{j}^{p}\in V^{p}$, denoted as $ATT_{kj}^{i}$ (*k*  =  1, 2, … , *h*), from the well‐trained model, and then identify species that highly contribute to each sample based on the attention value. Given that each attention head captures distinct relations on the heterogeneous graph in its individual channel, the *i^th^* species is of high‐contribution species to the *j^th^* sample as long as the attention of any of the heads $ATT_{kj}^{i}$ is higher than the threshold *thr_kj_*.

(17) $$thr_{kj}=\mathrm{mean}\left(A_{kj}\left[Q_{1},Q_{3}\right]\right)\;+z_{\alpha }\mathrm{sd}\left(A_{kj}\left[Q_{1},Q_{3}\right]\right)$$

where *A*_*k*,*j*_[*Q*_1_,*Q*_3_] represents the array of all non‐zero attention values between the quartile and the third quartile in the *k^th^* head attention of all species to the *j^th^* sample, and *z*_α_ is the confidence level. Here all attention in determining the threshold *thr_kj_* to mitigate the impact of outliers in attention values were not used. In this way, the species that have a high contribution to each sample could get.

Next, the microbial community associated with a certain cancer type was inferred by detecting species with consistently high contributions to samples with the cancer type. Specifically, when identifying the microbial community associated with the *c^th^* cancer type, the species with a high contribution to any sample with the *c^th^* cancer type was first collected. Then, for each species, the number of samples with the *c^th^* cancer type that each species significantly contributed was counted, denoted as $T_{c}^{i}$ for the *i^th^* species. Based on this, an approximate *p*‐value for each species (denoted as $P_{c}^{i}$ for the *i^th^* species) to determine whether the species is associated with the *c^th^* cancer type more often than expected purely by chance was assigned. The *p*‐value was shrunk using a method described in Section S2 (Supporting Information).

(18) $$P_{c}^{i}≅\sum_{t\;=T_{c}^{i}\;}^{U_{c}}\left(\begin{array}{c} U_{c}\\ t \end{array}\right)\times {\left[\frac{\left(\begin{array}{c} M-1\\ S_{c}^{max}-1 \end{array}\right)\;}{\left(\begin{array}{c} M\\ S_{c}^{max} \end{array}\right)}\right]}^{t}\times {\left[\frac{\left(\begin{array}{c} M-1\\ S_{c}^{min} \end{array}\right)\;}{\left(\begin{array}{c} M\\ S_{c}^{min} \end{array}\right)}\right]}^{U_{c}-t}$$

where *U_c_* is the number of samples with the *c^th^* cancer type. *M* is the number of all species. $S_{c}^{max}$ and $S_{c}^{min}$ are the sizes of the largest and smallest species sets that significantly contribute to the corresponding samples with the *c^th^* cancer type. The microbial community consists of species with $P_{c}^{i}<0.05$ is output as an inferred community associated with the *c^th^* cancer type.

### The Evaluation of Reproducibility Performance

To assess the reproducibility of identified cancer‐associated microbial characteristics, randomly subsampling a certain proportion (denoted as *r* of samples from the original dataset to form a “new” dataset was stratified. The random selection repeated *K* times and generated *K* datasets, denoted as $M_{k}^{r}$ (*k*  =  1, 2, … , *K*). Then, the method for cancer‐associated microbial characteristics identification was applied to $M_{k}^{r}$ (*k*  =  1, 2, … , *K*), respectively. The identified species of the *c^th^* cancer type was represented as $F_{kc}^{r}$ (*c*  =  1, 2, … , *X*). Then, for each cancer type, the Jaccard index was used to assess the similarity between the characteristics identified by each of the 2 datasets. The average of $\frac{K(K-1)}{2}$ pairwise similarities, *RJ_c_*, was used as the reproducibility index for the *c^th^* cancer type. The average of the reproducibility indexes across all cancer types, *RJ*, was used to assess the overall reproducibility of the identified characteristics, and a higher value corresponds to a higher reproducibility.

(19) $$Jaccard\;\left(U_{1},U_{2}\right)=\frac{U_{1}\cap U_{2}}{U_{1}\cup U_{2}}\;$$

(20) $$RJ_{c}=\frac{2\sum_{i=1}^{K}\sum_{j=i+1}^{K}Jaccard\left(F_{ic}^{r},\;F_{jc}^{r}\right)}{K\left(K-1\right)}$$

(21) $$RJ\;=\frac{\sum_{c=1}^{x}RJ_{c}}{X}\;$$

here, *K* = 50 and *r* ranging from 80% to 95% with a step of 5% to assess the reproducibility was set. The competing methods include a statistics‐based method, LEfSe, and a network‐based method, NetMoss, as well as the 6 machine learning methods (Lasso, Enet, RF, SVM, MIIDL, and PopPhy‐CNN). LEfSe and PopPhy‐CNN were implemented using the published source code. NetMoss was implemented using the R package NetMoss 0.1.0. Lasso, Enet, RF, and SVM were implemented by the MetAML software.^[^ ^32^ ^]^ MIIDL was implemented via the python package miidl 0.0.5. All of them were run on the Pitzer cluster of the Ohio Supercomputer Center with memory usage set to 300GB. The tunable parameters of each method using a grid search were optimized (Section S3, Supporting Information).

### Survival Analysis

Data on patient survival time was collected from TCGA‐CDR.^[^ ^41^ ^]^ When exploring the links between identified microbial communities and the survival time of patients, whether a given community was present within a sample was first determined. This was based on the relative abundance of microbial species. If all microbial species within the community were of non‐zero relative abundance within a sample, the sample was considered as with the community. Given the low biomass of intratumoral microbiome, there may be species with spurious zero abundance. Therefore, a community was presented within a sample as long as 80% of species in the community were of non‐zero abundance. Based on this, whether a given community was predictive of OS was explored, adjusting for the other communities as underlying confounding variables. By calculating the propensity score for each sample, paired sample groups were constructed, corresponding to groups with and without the community. This was conducted using the *R* package MatchIt 4.5.0 in *R* version 4.1.0. Kaplan‐Meier survival analysis was implemented to assess time‐to‐event distributions for OS, and the log‐rank test was used to assess the differences between the 2 groups.

### Mouse Studies

A total of 22 C57BL/6J mice were administered an antibiotic cocktail in their drinking water for 5 days to deplete the resident microbiome, followed by 2 days of regular water. This cycle was repeated 3 times in succession, first with a low‐absorption cocktail (ertapenem (1 mg mL^−1^; Merck and Co., Inc., Whitehouse Station, NJ, USA) Neomycin (1 mg mL^−1^; Fagron, Inc.), and Vancomycin (1 mg mL^−1^; Mylan Institutional LLC, Rockford, IL, USA)), then a high‐absorption cocktail (ampicillin (1 mg mL^−1^; Sandoz, Inc., Austria), cefoperazone (1 mg mL^−1^; Thermo scientific, USA), and clindamycin (1 mg mL^−1^; Major Pharmaceuticals, Inc., NJ, USA)), followed by the low‐absorption cocktail a second time. Three days before the end of the antibiotic course, a model system proceeded that faithfully replicates immune checkpoint responses in patients with microsatellite‐instability‐high tumors: the syngenetic, heterotopic mc38‐C57BL/6 system. Briefly, 1e6 mc38 cells are injected subcutaneously in the right flank. Following the antibiotic course, mice were gavaged with 200 µL stool slurry (1 g healthy donor stool in 5 mL de‐gassed phosphate‐buffered saline) with (*n* = 10) or without (*n* = 12) 1e7 CFU *B. massiliensis* strain FDAARGOS 3 days after cancer cell injection. Ten days after the start of tumor growth, mice were treated with either 5 mg kg^−1^ anti‐PD1 Ab (clone RMP1‐14; BioXCell, USA) or isotype control (IgG) (clone 2A3, BioXCell, USA). Tumor sizes were measured every 3 days by caliper. The experiment ended 21 days after mc38 injection and 6 tumor samples were randomly collected from mice gavaged with or without *B. massiliensis*.

To evaluate the presence of microbes in tumors, the tumor samples were sterilely dissected upon euthanasia. RNA was purified using an AllPrep kit (QIAGEN) following the manufacturer's instructions, with the addition of a bead‐beating lysis step. Specifically, tumors were minced by scalpel and then beaten in lysis buffer with 100 mg ceramic beads at 2000 rpm for 2 min on a Powerlyzer 24 (QIAGEN). Human and mouse RNA was depleted using a KAPA RNA Hyperprep kit (Roche). Samples were sequenced 2 × 150 nt on a NextSeq 2000 to a depth of 50 M reads and microbes were quantified using the {exotic} pipeline.^[^ ^12^ ^]^

### The Distribution of Attention Scores

For every head *h* (*i* = 1,…,*k*) of the multi‐head attention mechanism, the attention scores learned from each species to COAD samples were systematically ranked. This involved obtaining the *h* attention score ranks for each species *s* with respect to sample *p*, choosing the highest among them to represent the attention rank of species *s* to sample *p*. Subsequently, the Wasserstein distance as a metric to quantify the similarity in attention rank distribution across species belonging to the *Blautia* genus in relation to COAD samples was employed. The smaller the Wasserstein distance, the more similar the 2 distributions were. The Wasserstein distance between the attention rank distribution *v* of species *s_1_* to COAD samples and the attention rank distribution μ of species *s_2_* to COAD samples.

(22) $$W\left(x,y\right)=inf_{\pi \in \Gamma \left(x,y\right)}\;\int_{R\times R}\left|x-y\right|d\pi \left(x,y\right)$$

Which Γ(*x*, *y*) is the set of distributions on *R* × *R* whose marginal distributions are ν(*x*) and μ(*y*) on *R*.

### Statistical Analysis

Kaplan‐Meier survival analysis was implemented to assess time‐to‐event distributions for OS, and the log‐rank test was used to assess the differences between the 2 groups with and without the interested community. These were conducted using R package survival 3.1.12 in R version 4.1.0.

For functional analysis of host genes associated with microbial species, mRNA expression data of normal tissue and primary tumor samples with COAD in TCGA was searched. The DEGs between the 2 types of samples were identified using DESeq2 1.38.2 in R version 4.2.2 and filtered with *p*‐adjusted < 0.05 and the absolute value of log2FoldChange > 1. Then, the Spearman correlation coefficient was calculated between the abundance of microbial species and the expression of DEGs by Scipy 1.10.0 in python version 3.9.13, and enrichment analysis for significantly negatively correlated genes by KOBAS.^[^ ^71^ ^]^ Multiple testing correction was implemented using the Benjamini‐Hochberg procedure.

For mouse studies analysis, considering the repeated measurements for each mouse, tumor size data was analyzed using two‐way repeated measures ANOVA for mice before treatment to estimate the effects of *B. massiliensis*. A longitudinal mixed‐effects model for post‐treatment data was built followed by Sidak's correction for multiple comparisons. The statistical test used, and P values are indicated in each figure legend. *p* values less than 0.05 were considered statistically significant. *p < 0.05, **p < 0.01, and ***p < 0.001.

## Conflict of Interest

The authors declare no conflict of interest.

## Supporting information

Supporting information

## Data Availability

The data that support the findings of this study are openly available in the TCMA database at https://doi.org/10.7924/r4rn36833, reference number 10.
