# Supplementary material for: An Explainable Graph Neural Framework to Identify Cancer‐Associated Intratumoral Microbial Communities
Source: Adv Sci (Weinh). 2024 Sep 3;11(41):2403393. doi: 10.1002/advs.202403393 (PMC11538693; doi:10.1002/advs.202403393)
Supplement: Supplementary file 1 — Supporting information [file ADVS-11-2403393-s001.docx]

Supporting Information

An Explainable Graph Neural Framework to Identify Cancer-Associated Intratumoral Microbial Communities

Zhaoqian Liu^#^, Yuhan Sun^#^, Yingjie Li^#^, Anjun Ma, Nyelia F. Willaims, Shiva Jahanbahkshi, Rebecca Hoyd, Xiaoying Wang, Shiqi Zhang, Jiangjiang Zhu, Dong Xu, Daniel Spakowicz^*^, Qin Ma^*^, Bingqiang Liu^*^

^#^ These authors contributed equally to this work.

^*^ To whom correspondence should be addressed: Dr. Qin Ma, qin.ma@osumc.edu; Dr. Bingqiang Liu, bingqiang@sdu.edu.cn; Dr. Daniel Spakowicz, daniel.spakowicz@osumc.edu.

This file includes:

Figure S1 The heatmap of correlation between the expression of DEGs and the abundance of microbial species.

Figure S2 The association between microbial species from MICAH and host gene expression.

Figure S3 The distribution of attention score rank of *Blautia obeum* and other species belonging to Blautia in COAD samples.

Figure S4 Relative abundance of *Blautia* in tumor tissues.

Figure S5 Details of attention mechanism for node embedding update.

Table S1. The reproducibility performance on the five fungi datasets.

Table S2. The microbial communities identified by MICAH.

Table S3. Intratumoral microbial species associated with the five cancer types from peryton.

Table S4. Body site attribution for cancer types from Sourcetracker2.Table S5. The species contributed by attention and both phylogenetic and metabolic relationships in MICAH.

Table S6. Main effects on tumor volume before the immunotherapy.

Table S7. Longitudinal mixed-effects model post-immunotherapy.

Table S8. ANOVA table of the Longitudinal mixed-effects model post immunotherapy.

Table S9. The number of samples of the five cancer types from TCMA.

Supplementary Section S1. The construction of phylogenetic links at different phylogenetic levels.

Supplementary Section S2. Determining the *p*-value for a species to a cancer type.

Supplementary Section S3. Parameter optimization of machine learning methods.

**

**


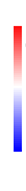


-0.2

+0.2

**Figure S1 The heatmap of the correlation between the expression of DEGs and the abundance of microbial species.** Each row is a microbial species identified by MICAH, each column is a DEG, and each cell shows the Spearman correlation coefficient between a species and a DEG.

**Note**: The microbial species from top to bottom: Fusobacterium periodonticum, [Ruminococcus] gnavus, Bacteroides sp. UW, Bacteroides fragilis, Bacteroides ovatus, Enterocloster clostridioformis, Bacteroides xylanisolvens, Bacteroides eggerthii, Phocaeicola vulgatus, Parabacteroides merdae, Faecalibacterium prausnitzii, Alistipes shahii, Agathobacter rectalis, Eubacterium ventriosum, Mediterraneibacter faecis, Anaerobutyricum hallii, Phocaeicola massiliensis, Bacteroides thetaiotaomicron, Anaerotruncus colihominis, [Clostridium] leptum, Bacteroides stercoris, Phocaeicola dorei, Bacteroides uniformis, Bacteroides intestinalis, Bacteroides caccae, Parabacteroides distasonis, Veillonella dispar, Veillonella parvula, Veillonella sp. 3_1_44, Veillonella sp. 6_1_27, Akkermansia muciniphila, [Bacteroides] pectinophilus, Pseudoflavonifractor

capillosus, Roseburia intestinalis, Roseburia faecis, Roseburia inulinivorans, Holdemanella biformis, [Ruminococcus] lactaris, Subdoligranulum variabile, Butyrivibrio crossotus, Lachnospira eligens, Oscillibacter sp. ER4, Collinsella aerofaciens, Dorea longicatena, Coprococcus comes, Solobacterium moorei, Fusobacterium sp. oral taxon 370, Fusobacterium sp. CM21, Fusobacterium nucleatum, Phocaeicola coprocola, Phocaeicola plebeius, Holdemania filiformis, Butyricimonas virosa, Bacteroides finegoldii, Haemophilus parainfluenzae, [Clostridium] symbiosum, Enterocloster asparagiformis, Parabacteroides johnsonii, Alistipes putredinis, Bilophila wadsworthia, Odoribacter splanchnicus, Barnesiella intestinihominis, Blautia hansenii, Parabacteroides timonensis, Segatella copri, Phocaeicola coprophilus, Phocaeicola salanitronis, Blautia obeum, Lachnospiraceae bacterium 2_1_58FAA, [Clostridium] nexile, Dorea formicigenerans, [Ruminococcus] torques, Hungatella hathewayi, Enterocloster bolteae, [Clostridium] scindens, Bacteroides helcogenes, Bacteroides cellulosilyticus, Marvinbryantia formatexigens.

DEGs from left to right: UROC1, H2AC12, H4C3, PLA2G4B, SNHG20, LOC124909475, ATP6V1FNB, NT5DC4, NPIPB4, CRNDE, LOC101927480, ST20, TTC34, PCAT2, LRRC9, TSEN2, ADAT2, AARSD1, MSH5, AHSA2P, EIF4A1, H1-4, H1-5, H1-3, SNORD15B, KYAT1, SCLY, H4C5, H4C4, H2BC17, LRP8, ABHD16A, SNORD72, PLAC4, TATDN1P1, SNORA3B, MALAT1, SNORA54, H2BC10, C3P1, LOC124901427, RPL6P13, FANCG, NCAPD2P1, RPL10P6, CCL20, TNNI3, SLC17A9, CBARP, KREMEN2, BSG-AS1, MSX1, HTR1D, NQO2, MCIDAS, TP73, MEGF6, PCSK9, RELT, TNFSF9, B3GNT4, FOSL1, MROH6, LOC100288175, MIOX, GPR3, BHLHE40, LIF, PLK3, MYEOV, SLC4A11, CYSRT1, LOC254896, WDR62, UHRF1, PANO1, LIF-AS2, TNNT1, GRIN2D, ALDOC, PHLDA2, MFSD12, UBE2S, MAD2L2, PLEKHN1, TNFRSF12A, KLHL17, TEAD4, PIERCE1, HAGHL, WDR54, F12, CCNO, ZNF511, NXPH4, ZIC5, ZIC2, VSNL1, CYP4X1, ERFE, TPRXL, CASC8, FEZF1-AS1, FEZF1, PLA2G3, PAX9, PNPLA3, IRX3, CCNP, CLDN14, TFAP2C, PELP1-DT, EGFL6, WNT2, PPEF1, RPL5P4, SMYD3, CTPS1, URB2, AJUBA, GRB10, SYT12, MUC16, SMCO2, FRMD5, SEC14L2, MIR34AHG, LNCTAM34A, BMAL2, EIF5A2, HOMER1, NFXL1, FAM216A, MATCAP2, OSBPL3, COA1, SNORD100, PRNCR1, MIR4435-2HG, DIABLO, GNA15-DT, ITGB1-DT, PPIAP51, LINC02577, GRHL1, CYTOR, C2orf27A, NPSR1-AS1, NPSR1, CHST4, SP8, IL36RN, CACNG8, PPP2R5CP, HAUS7, SCART1, C8orf74, VAC14-AS1, PCNX2, MAP3K20-AS1, STX16-NPEPL1, TRIM72, SLCO1B3, SLCO1B1, CHKB-CPT1B, SNORD99, PABPN1, ZC3HAV1L, LOC728554, SNHG10, RPL13P6, RPS27AP12, G3BP1P1, MTHFD1P1, NDUFA9P1, MPP3, RPL17P50, SNORD12C, SLC34A3, RDH16, AFAP1-AS1, GREP1, ONECUT2, BAAT, SPATA12, SPRY4-AS1, EXTL3-AS1, LINC01913, NAP1L4P3, SNORD17, RNU4-1, RNU4-2, RNY3, RN7SKP9, SCARNA9, SCARNA9L, SNORA20, RNU6ATAC, SNORD13, LOC124904619, SCARNA7, RPL31P19, RPL11P3, LOC107984970, CRLS1, TWIST1, PDPN, SPARC, EPYC, CTHRC1, PLPP4, COMP, THBS2, COL1A1, BGN, ADAMTS2, CNIH3, BEAN1, CPZ, PIANP, ADAMTSL1, CALB1, LUCAT1, STC1, C5orf46, SERPINE1, LINC02257, XIRP1, OLR1, ADAM12, SULF1, CORIN, TNFSF4, INHBA, CCN4, NOX4, FAP, COL10A1, ADAMTS12, ANOS1, COL22A1, COL11A1, RFX8, RPSAP52, ADAMTS4, ZNF469, SLC27A5, SPNS3, EIF4EBP1, CCNE1, GET4, ZNF771, RPL34P18, RPL10P13, RPL31P49, UBE2NL, RPL18P8, RPL8P4, RPS4XP3, RPS6P10, RPS23P8, RPS18P5, RPS9P2, RPS15P4, RPS13P2, RPL3P9, RPS10P2, RPL26, KISS1R, PPP1R14B-AS1, PSMD9, RNF183, CAPN12, HES4, LAIR2, PRSS22, CHAC1, RBM39P1, RPL31P2, RPS8P9, RPL19P6, RPL23AP65, RPL23P6, RPS17P16, RPLP0P4, RPL34P27, ANP32BP1, HSP90B2P, TCP1P3, PARP1P1, RPL5P8, LOC100420423, RPL26P4, HSPD1P5, RPL7P33, LOC729966, NAP1L1P3, LOC149844, SETSIP, IFITM9P, RPL31P4, KRT18P18, RPL7P10, AKR1C4, SERPINA4, ABCC2, RXRG, P2RX2, CREB3L3, LINC01645, TRPV3, APOA2, F2, LOC102724908, TRIM71, CTNND2, CPNE5, TNFRSF13B, IRF4, TNFRSF17, IGHV3-11, C2orf88, IGF2BP3, CCN6, HNRNPD-DT, ASIC4, CCDC198, IRX5, HDAC10, TMEM74B, DTD1, DSC3, LINC02009, NPC1L1, DSG4, DHRS2, ATP6V1C2, MYBPHL, LPO, FXYD4, CWH43, NR3C2, NXPE1, B3GALT1, CA12, PADI2, EPIC1, SAMMSON, CPB2, FAR2P1, LOC124903770, LOC101926964, NTMT2, NEUROG2, CALCA, ISM2, LINC01694, WT1, EN2, SNHG15, SMKR1, KAT2A, PDCD5, ACTR3B, NSUN5P1, TAFAZZIN, POLR1C, LYRM4, MRGBP, TOMM34, NAA80, DXO, RTKN, ETV4, CMTM8, WDR74, NTMT1, DPH7, TRMT1, ZMYND19, WRAP73, MRTO4, S100A11, PCAT6, PPP2R3B, CBX8, BYSL, E2F1, RRP9, ANKRD16, NOP16, RUVBL1, MRPL17, EXOSC7, ATRIP, ATRIP-TREX1, NPRL3, SPINDOC, MARCHF9, HAUS5, TWNK, TFAP4, RHEBL1, C2, KIF9, GNL3, UBE2T, CMBL, EXOSC2, ENTR1, GALNT12, ANKS6, QSOX2, UBXN11, TROAP, PIF1, ADAMTSL5, CCDC78, SLC25A29, IZUMO4, PBX4, NOL3, EME2, WDR90, HSF4, ANKRD13D, FBXL8, CCDC57, ANKS3, NUP153-AS1, PAK6-AS1, JAG2, P3H1, CHPF, LBX2, SFXN3, SPTBN2, TMEM120B, RRP12, AMPD2, ATXN7L2, RHBDF2, AGAP3, S100A2, LINC00659, SLCO4A1, FNDC11, RELL2, WFDC3, CDH3, TMEM105, DUSP2, TPD52L1, AUNIP, CDC25B, IMPDH1, CBX4, CAPN10, ST7-AS1, TRIP13, RFC4, LINC03040, FAM53A, SPATA3-AS1, ARG2, EME1, DDX11-AS1, MFAP2, MMP11, CPXM1, THY1, FKBP10, CILP2, AQP5, SMPD1, PODNL1, PDX1, HOXB8, SHB, OTUB2, COL9A3, SLCO4A1-AS1, TRIM27, KIAA1549, RP9P, STX1A, KRT15, IRAK2, CAMK2N1, MUC13, SLC6A20, LOC105376380, CASZ1, ADCY9, NDST1, NHSL1, PLXNA2, TSHZ1, TNFSF10, OPTN, BCAR3, MGLL, CORO2A, CGN, VDR, FUCA1, TMPRSS2, LOC124905021, SULT1A1, CARD14, LIME1, LRRC75A, TP53INP2, TLCD2, GTPBP4, WASHC2C, WASHC2A, SH3KBP1, PLEKHA7, BRPF3, ARHGAP17, ATP6V1E2, SPATA24, PPIAP45, PLD1, TEP1, ANK3, RBM47, PPARGC1B, KBTBD11, SLC41A2, MGAT4A, SLC20A1, BMAL1, MIDEAS, LUZP1, NCOA1, MTF1, UBR2, MOB3B, SLC35G1, RAVER2, ANKRD13A, MXD1, LRRC1, LGR4, ZZEF1, KIF13B, PTK2B, TRANK1, MARCHF8, ZMIZ1, CCDC68, TNFRSF10B, DPF3, HK2, IL18BP, PCOTH, PSD2, RAET1K, RPL7AP39, SCIRT, P2RY1, CFAP45, ZNF692, CSNK1E, CLBA1, KLHL2P1, ZNF239, CCNB1IP1, MTHFD2, TGIF1, CCDC59, REXO2, XPO5, POLR1B, RCL1, PACC1, RPP40, PLD6, MTHFD1L, TXNRD3, PHKA1, SETD6, CCND1, FAM86DP, ACP6, LOC155060, AHCYL2, MYO1A, KIAA0513, SLC22A23, MYO1D, MLXIP, ASAP3, MAOA, PLCG2, CD22, LY9, IL6R, LINC00092, LOC105376159, IL16, PPP1R16B, GIMAP1, ADORA3, CD180, RCSD1, TRBV7-3, GIMAP7, P2RY8, SPIB, IRAG2, NUGGC, TENT5C, FRMD3, RAB27A, FAM107B, CASP10, GBP3, LIMA1, CAST, GPAT3, ABHD3, STYK1, ASPA, LINC02884, GRIN3A, AP3S2, OXTR, NEB, ACP4, GRIN2B, KRT18P4, SLC22A5, KIF16B, SLC36A1, ZSWIM6, KCNJ14, MC1R, LRRC46, WNT3, LOC101929777, C9orf43, COL27A1, LINC02263, SH3PXD2A-AS1, SIX4, ADAMTS6, RNF152, ESM1, H3C12, MYOM1, DAAM2, SLIT3, CNNM2, ADCY5, CDKN2B, GLDN, SYNC, AOC3, TNS1, NCAM1, MYOCD, SORBS1, CNTN4, BVES, MYLK, MSRB3, PKNOX2, SCN2B, CPXM2, SVIL, RNASEL, PTPN21, NBPF25P, PLPP3, FNBP1, PBLD, ACOX1, FMO4, HSD17B6, SUGCT, UNC5C, CPNE8, PDE8A, CLMN, TMEM131, NBPF1, PEX26, KIAA1671, PHLPP2, MIER3, MAGI3, BCL2L11, EGFR, CBFB, MARCKS, CAB39, SLC44A1, SPATS2L, LGALSL, CIPC, WASL, PDCD6IP, SH3BGRL2, MTM1, TRAK2, ABHD5, RMDN2, LITAF, INAFM2, CALCOCO2, WIPF2, PLCE1, ARPIN, GNPTAB, CCNYL1, USP38, MACIR, NIPAL1, SLC35D1, RIOK3, TGFA, UGP2, NCOA4, SCP2, RAP1A, STX12, ETFDH, ATP8B1, VPS4B, RCAN1, DNAI4, PLS1, GNG12, ELAVL4, PLSCR4, KAT2B, CLIC5, SUSD6, PPP1R12B, MBNL1-AS1, DSC2, VCL, EIF4E3, ATP2B4, ARRDC4, SPPL2A, ASB7, RHOU, RFK, BTBD3, LEXM, ATP11A, DCUN1D2, CLEC9A, PKHD1L1, AQP7, AFF3, ALOXE3, VNN1, VNN3P, PGLYRP4, LINC02882, LINC02341, CXCL17, LINC01630, STC2, ERVMER34-1, TBC1D30, STRIP2, SNHG1, RPS17, SNHG3, IGHV3OR16-9, SLC26A2, PDE6A, SEMA6A, LOC124907763, HAPLN1, SLC46A3, HHLA2, TMEM253, C4orf19, CCL28, MOGAT2, ABCC13, TTLL6, GPR157, ZNF251, DCAF13, TGIF2, TCFL5, LINC03011, MHENCR, PAXIP1-DT, CEP72, STPG4, MATN1-AS1, LOC105375421, KCNH8, NFE2L3, CFAP92, CDK11A, GTF2H4, SCAT8, SHH, TG, NPFFR1, LRRC36, ARID3A, ADSL, SLC11A2, FAM227A, ARHGAP27P1-BPTFP1-KPNA2P3, TSACC, ZFAS1, SNHG17, MINDY4, RPL36A, MIR17HG, PVT1, SNORA33, SNORD46, MACC1, NKRF, SLC19A2, C5orf34, C2orf15, WDR5B, TIGD1, RFXAP, EXOSC3, TAMM41, SOX4, SLC16A1-AS1, LOC389906, FAM239C, TSPAN5, RAD54B, DDIAS, ZNF749, SNHG16, UBE3D, RAD54L, PSMC3IP, ORC6, TMEM41A, CCT6B, PTRH2, RCN1, B3GNTL1, AGAP9, NEBL, ENC1, TNFSF15, TDP2, NEDD4L, SH3TC2-DT, SLC51A, TAT, LMNTD2-AS1, RTEL1-TNFRSF6B, LTB4R, SNHG12, CHKB, KCP, CCNL2, RPS6KL1, ANKRD39, PSMG4, VWA2, LINC01605, BBOX1-AS1, LOC105375790, CPA5, RP1, MYBPC3, PILRB, CDK5RAP3, DHPS, SNORA73B, C20orf144, PASK, LUC7L, XPOTP1, ULBP3, VEGFA, LINC01270, LOC105371956, KIF26B, SNORD69, RPL10AP1, ELOA-AS1, NOP2, LOC101927401, SULT1C2, LEF1, ESAM-AS1, ZMIZ1-AS1, SUGCT-AS1, SLC7A11, LOC157273, LOC101929128, ZNF121, XRCC2, CCDC150, MET, TRMT11, TEX10, THUMPD2, WDR43, WDR75, EEF1E1, DCUN1D5, TAF1D, PMAIP1, PPAT, NAP1L1, MSH5-SAPCD1, SAPCD1, NPIPA1, NPIPA2, TLX1, MST1, RPP21, TRIM39-RPP21, KRTCAP2, NPIPP1, PABPC1L, CYP2D8P, LINC01132, SUPT4H1, TSPOAP1-AS1, CD44, CCDC192, KRT18P15, LCAL1, LINC01807, LINC01748, LINC01730, GARIN2, LOC124902439, CLCNKB, HBB, EYA2, CYP26B1, SH2D7, NANOS3, MAT1A, LHFPL7, TESC, FAM166C, PGC, CLDN2, AQP5-AS1, PRSS33, C6orf15, EDAR, FOXQ1, LINC00460, KRT6B, KRT75, KLK6, DKK4, FGF20, SP5, LINC01124, NOTUM, DPEP1, SLC6A14, TGFBI, BFSP1, RPSAP54, MMP7, RPLP0P2, C2CD4A, TCN1, C2CD4B, NKX2-1, RPSAP71, TMEM158, DMRTA2, SOX14, PLIN1, LOC643015, LEMD1-AS1, CEMIP, HNRNPA1P27, SLC30A10, SCARA5, GLP2R, ENHO, BEST4, TMIGD1, LYPD8, GUCA2B, CA4, MS4A12, CLCA4, CHP2, AQP8, SCNN1B, CLEC3B, DUSP26, CHGB, FEV, CHGA, ABCG2, LIPC, RHEX, CPM, TPH1, SLCO4C1, SCGN, ACKR2, LYVE1, CD36, CCR2, MMRN1, KRT1, VSTM2A, TEX11, SELENOP, OBP2B, LINC01411, BMP7, SLC35D3, LGR5, LINC01996, IGFL4, CRAT37, IGFBP1, RPS15AP17, MRPL38, INO80B, CFAP119, CYB561D2, IPO4, ACY1, MTHFS, UBD, PGK1P2, EIF3KP2, LOC149935, RPL5P18, ALDH7A1P1, LOC317727, RPL31P61, RPS4XP2, RPS4XP8, AGAP2-AS1, TPGS1, NME2, TAX1BP3, CTU1, PDF, NUDT3, POLR2I, RAB5IF, SRXN1, RPS20P6, RPS18P2, RPS19P7, RPL18P13, RPL18P12, TECRP2, RPL13P2, RPL18P4, RPS5P8, RPL10AP12, SSTR5, RPL27P4, MIF, BCKDHA, RPL13P12, GNG10, ARL2BP, SPRR1A, SPRR1B, SPRR3, CCL3L1, CCL3L3, APOBEC3A, APOBEC3A_B, TRGV4, PCOLCE2, CSF3, CSF2, MMP3, MMP1, IL11, CXCL5, IL24, SERPINB7, PPBP, MMP10, B4GALNT2, BTNL8, EDN3, OTOP2, CA2, B3GNT7, PYY, KRT8P36, MCEMP1, IGFL1, RERGL, SIGLEC11, LGALS12, PRSS56, WNT11, EVA1A-AS, SERPIND1, TMX2-CTNND1, C1orf105, POU5F1B, PCAT1, LOC105375751, SRPX2, LY6G6F, TUBAP13, AMELX, KRT23, LY6G6D, LY6G6F-LY6G6D, LY6G6F-LY6G6D, LINC01811, TDGF1, ACSL6, LOC105372710, PAH, SLC22A11, CKMT2, CYP24A1, RDH12, LINC00858, FER1L4, LOC389602, GABRE, SPNS1, FKBP9P1, STK31, F2RL2, TCF24, WDR35-DT, TNFSF11, APELA, LINC01978, LINC01273, PCID2, MIPEPP3, SSC4D, ATG9B, ASB9, CPNE9, GDPD5, ISX, NKPD1, LOC105370027, PMEPA1, NKILA, LEF1-AS1, GRP, SHISA2, MATN3, ALKAL1, SLC2A12, MOCS1, CDIPTOSP, DLX4, PLEKHA8P1, RNF43, DTNB, ARHGAP44, CARMIL3, SLC6A6, NFASC, CBY2, CELSR1, URAHP, SLC2A4, CADPS, PAQR8, TCF7, MFSD4A, USP2, ENPP6, ADAMDEC1, EPHA7, EDIL3, ITGA8, METTL7A, PRKACB, NAAA, NR5A2, C7orf31, GPR19, APPL2, PDCD4, GPD1L, PRR5L, RBKS, CAT, LARGE1, GRAMD2B, PIP5K1B, SUCLG2, HIGD1A, TMPRSS5, EXOSC8, POLR1D, RFC3, SKA3, NUFIP1, EBPL, FAM83D, GINS1, SCD, SPATA33, TMEM147-AS1, LINC01133, TRMT6, NANP, RNF32, SLC16A9, ENTPD5, EPB41L4B, BMX, SNTB1, IL20RA, STAG2-AS1, DSCC1, PRMT3, CMSS1, CSE1L, PUS7, TTC26, XPOT, C12orf73, DUS4L, TMEM97, LDLRAD3, DNMT3B, TFAP2A-AS2, SYPL1P2, ABHD1, SEMA6A-AS2, SEMA6D, TMEM236, LRRC19, DDN-AS1, PAQR5, XPNPEP3, AQP6, SLC22A3, MEX3A, LINC02418, LINC01979, BTBD16, SLC2A1-DT, CSTL1, PTGES2-AS1, SPTBN5, LOC105370792, BLACAT1, PHLDA1, ADAM20P3, SH3TC2, TAS2R38, PTPMT1, RPS3AP49, RPL5P12, NPW, NPIPB5, JTB, RANGRF, PET100, RPS10, TOMM5, MRPS24, RPS27AP5, ABCB6, CNPY2, UCHL3, LINC01836, GNG4, KCNK9, UCA1, FOLR1, ULBP1, PPM1H, FOXP4-AS1, FUT1, IGFL2-AS1, ADH1B, TMEM100, ABCA8, ECRG4, PLP1, BMP3, CA1, CDKN2B-AS1, PRIMA1, DMRTA1, RBFOX3, PRKG2, CXCL12, PDK4, VEGFD, CST4, CST1, CST2, RSPH14, LINC01315, ASCL2, LZTS3, LAPTM4B, CPNE1, AHCY, GLYATL1, SLC30A2, TM4SF1-AS1, NKD2, TH, AZGP1, CLDN9, MMP20, AREG, EREG, TRPM6, UGT2A3, SLC26A3, CLDN1, CLDN16, LOC440742, GBX2, AXIN2, ZNRF3, LINC01234, HOGA1, MAGEB17, SLC13A3, XKRX, STRA6, CFAP251, SALL4, LOC107985323, LINC01101, SLC17A4, SULT1B1, BRSK2, FGF19, PDIA2, LRRN4, CEL, DUSP15, IGSF23, CELP, UNC93A, CYP2W1, EVA1A, PALS2, SLC39A10, TM4SF19, TMEM40, SLC5A4-AS1, DUXAP10, DUXAP8, DUXAP9, NXPH1, PURPL, FIRRE, LINC02985, TUBB3, ELF5, HS6ST2, ANKRD1, TSPEAR, TSPEAR-AS1, RPS3P7, COMMD7, SNORD104, RPS2P20, RN7SL3, SNORA74A, RAET1L, IL1A, KRT7-AS, LINC01433, GNMT, CNPY3-GNMT, LOC101059948, SPOUT1, CXCL1, CXCL3, CXCL2, IL23A, ABCA12, LINC02154, LINC01303, SPRR2D, SPRR2A, FOXD1, GAD1, CEACAM18, TNRC18P2, FAM157A, PPFIA4, HES7, IGFL2, DUSP4, GAPLINC, CA9, TFAP2A, KIR2DL4, DLGAP1-AS5, UGDH, FGFR2, OTX1, PRKCG, MUC6, SBSN, KRT5, PAEP, KLK10, WNT7B, CLDN18, DMBX1, MIR31HG, C10orf55, RGS16, DUSP10, SLC9A7, RAD51AP2, LRRC73, NDUFA4L2, GJB4, GJB5, ULBP2, HNRNPA1P21, FOXP3, SPOCD1, CTLA4, CHI3L1, LINC02345, MMP12, IBSP, MMP13, LINC01614, CLEC5A, KCNJ15, SPP1, SLC11A1, TREM1, HAMP, SLAMF9, DCSTAMP, SSTR3, PLCL2, GBP2, CCL23, LILRB5, CD209, C16orf54, SECTM1, GLIPR2, SULT1A2, FLVCR2-AS1, EPB41L3, LRP2, SLC4A4, TRIM40, ADTRP, B3GALT5, MEP1B, ALPI, ANPEP, MSLNL, KLK7, KLK8, HABP2, SLC34A2, PLSCR3, PIRT, TAGLN3, COL7A1, UCN2, ACAN, LINC01615, LZTS1, PLXDC1, C2orf81, HCRT, GNG7, MAL, GFRA2, CD163L1, BMP2, RGS9, GPR37L1, HMCN2, PCSK5, NTN1, SLC9A3, DHRS11, CLCN2, UGT1A1, WASIR2, CHP1, ARHGEF37, RNFT2, S100A1, SOX9, CELSR3, CFAP157, ACACB, ZNF575, FBXL22, WDR72, LINC01833, SIX3, EGR4, ONECUT3, MEX3D, TOMM40P4, FGFRL1, UGT2B15, UGT2B17, SIGLEC15, GRIK5, PLIN4, PTRH1, TNS4, GSDMA, APOBR, CES3, CORO2B, PRPH, C7, SCG2, NSG2, SLC22A18AS, MALL, CSRP1, CAPN2, FLNC, TLN1, FLNB, LRRN2, TENT5B, PLAC9, DAND5, TOM1L2, ECE1, ARHGEF18, SLC44A4, SCAMP2, SESN2, IGSF9, ZBTB7B, GNA11, SLC9A1, MISP, FBLIM1, ZER1, SORD, DHDDS, STPG1, MARVELD3, GALM, HADHB, HADHA, PAFAH2, TRAF3IP2, NAT1, SQOR, RETSAT, PTPRH, TSPAN1, ACADS, CAPN5, SLC25A23, C1orf226, LAP3, LETM1, CASP7, SHROOM3, PKP2, CYP27B1, MPC1, CNN2, CNNM4, DENND1C, NFE2L1, MAST2, GLTP, PACSIN2, GRAMD4, GAS8, PRDX6, SPECC1L, CTDSP2, ANO10, GBA1, MYORG, TMEM127, STIM1, EPAS1, LRRC66, NEO1, IQGAP2, FAM161B, EHHADH, TMEM30B, SOWAHB, SMPDL3A, PAPSS2, F2RL1, IDH3A, GOLM1, CTSS, DECR1, SIGLEC8, LIPH, ENDOD1, MBOAT1, IGSF3, SDCBP2, SLCO2A1, RDH5, TUBAL3, BTNL3, CDHR2, SMIM6, UNC5CL, TMEM72, TSPAN7, CA7, GDPD2, PARM1, VAT1L, PEX11A, CPT1A, ABHD6, CEACAM1, VWA5A, SMOX, PCED1A, GABRD, GRAMD1A, FTSJ1, ACBD6, LHX4-AS1, SULT2B1, SNHG32, ZP3, SLC6A9, GDPD3, KRT20, TMEM45B, ACAA2, SIAE, CPT2, LPCAT3, CLU, SLC25A20, PLPP1, DENND2A, ACVRL1, PLCD1, C1orf210, TTC22, TESK2, CCNJL, C1orf115, TEF, CBX7, HMOX1, VSIR, PTGDR2, LGALS4, HSD11B2, GPA33, TMEM171, KLF4, PGM1, CR2, BRINP3, TINCR, GPR15, IGHA2, ADH1C, DNASE1L3, AMPD1, JCHAIN, FCRLA, IGKV2-24, IGKV2-30, IGHV3-72, IGHV3-74, IGHV3-7, IGHV3-15, BCAS1, TMEM37, PLAC8, DHRS9, AKR1B10, SLC51B, CEACAM7, MADCAM1, P2RX1, CCL19, CD177, LINC00974, DAO, PKIB, WSCD1, CLDN23, NKX2-3, NAALADL1, SLC25A34, CES2, TMEM82, GBA3, HSD17B2, GUCA2A, UGT1A8, UGT1A10, CCBE1, LPAR1, DPP6, GAP43, SRPX, TACR2, PSD, CADM3, BMP6, MAMDC2, SFRP1, PGM5, BCHE, OPN3, REEP2, ITIH5, TCF21, PTN, TMEM35A, HPSE2, FENDRR, NAP1L2, ATP1B2, GSTM5, GDNF, GFRA1, GREM2, STMN2, RCAN2, FAM107A, FHL1, CAVIN2, NEGR1, SYNPO, PDZD4, TPM2, MYL9, TAGLN, C4A, LOC110384692, PDE2A, NPR1, SEMA3G, ITGA7, POPDC2, KANK2, PI16, ASB2, HPGDS, ANO5, LOC102723370, ATP1A2, SCUBE2, CRYAB, MFAP4, RBPMS2, LMOD1, DNAJB5, DACT3, CYS1, MAP6, FBLN5, CLIP3, TNXB, LINC02984, GNAO1, SHISAL1, PYGM, JPH2, LDB3, MYH11, SYNPO2, SYNM, ADAMTSL3, ACTG2, CNN1, HSPB7, KCNMB1, HAND2, CASQ2, HSPB8, HAND2-AS1, JPH4, SALL2, RPS15P2, EIF4A1P10, RPL4P4, RPL4P5, RPS10P26, RPS4XP20, RPL10P9, RPS15P5, RPS2P48, HSP90AB3P, RPSAP46, NACA3P, RPS7P1, RPL10AP6, HNRNPKP4, RPL3P2, RPS9P4, RPLP0P6, RPS3P6, EEF1DP1, RPS3P3, RPS16P7, RPL18P3, RPS3P4, RPL5P1, RPL5P22, RPL3P7, KRT19P1, RPL26P32, RPLP0P5, RPS11P5, RPS15AP19, NACA2, RPL32P18, RPS6P25, RPS6P26, RPL5P17, RPS8P3, LOC344967, ATP5F1AP2, HSP90B3P, FKBP4P6, EIF4A1P7, RPL7AP4, RPL29P2, RPL5P5, HSPA8P7, EEF1DP5, SRIP3, EIF3IP1, TUFMP1, RPS13P8, RPL27P11, RPS19P1, RPL8P3, LOC440311, RPL28P5, ACTN4P1, RPL27P1, RPS19P2, RACK1P3, CCT6P2, RPL34P5, CCT7P1, NDUFA13, MRPL2, LOC124901186, LOC124907760, SNHG25, RPL34P31, PRC1, RACK1P1, EIF3EP3, PABPC3, RPS15AP24, RPS5P6, RPL11P2, RPL36P12, TRPV4, MMP9, SPHK1, PLAU, LOXL2, PLAAT2, GPX3, IGFBP6, CDKN2A, PANX2, MUC5AC, FAM131C, NMU, SYT8, MAPK15, NDRG1, MDFI, TIMP1, KRT17, KRT7, DSG3, KRT6A, KRT16, C9orf163, DNASE1L2, GOLGA7B, PLEKHG4, MRI1, LRRC8E, UCN, TMPRSS13, PKP1, RAB36, FABP6, ASGR1, STBD1, RIPPLY3, MSX2, KLK11, PITX1, NT5DC2, LDHD, LYPD3, PGGHG, LEMD1, SERPINB5, TMPRSS3, GABRP, LINC01709, TACSTD2, APLN, FJX1, KRT80, TRIM29, IL17C, ALDH3B2, STYXL2, ASCL5, MS4A15, DLX6, CAMKV, MAGEA6, MAGEA3, MAGEA12, AHSG, AFP, FGB, FGA, ZDHHC8BP, CXCL6, LINC01426, HPGD, EPHX4, LINC02253, ERICH2, SCAT1, ERICH2-DT, PIWIL1, SIM2, GLS2, MXI1, CITED2, TXNIP, DDX60, RELL1, SMIM14, ABCD3, CDC14A, TLR3, ABCB11, EMP1, TMCC3, CRACD, EML1, LIFR, ANK2, A2M, JAM2, SPARCL1, CCR8, PRKCB, FGL2, SLC9A9, ABI3BP, ARHGAP20, GIMAP8, NIBAN1, SHE, GCNT2, P2RY14, SCN9A, CFL2, CAV1, TMEM220, SLC17A7, CCDC69, TMEM140, ITM2A, POGLUT2, NEURL1B, PTGS1, RSAD2, ENPP2, MPEG1, GIMAP6, DNAH2, PPM1N, TBX15, PRECSIT, DOK7, LINC00239, ANXA3, PERP, GTF3A, SLC25A15, ENOPH1, BOD1, MRPS23, CDCA7, PNO1, POLB, TEX30, NTAQ1, NUDCD1, SQLE, KCNE3, CTSH, PSPH, DKC1, UBE2C, TPD52L2, NELFCD, RAE1, ACTR5, UTP4, WDR77, MNX1-AS1, MALSU1, RRP7BP, ACAT1, HILPDA, HSPE1, SNHG11, SLC5A6, RNASEH1-DT, NAT9, PDRG1, TOP1MT, RRS1, IQANK1, TRMT112, RNF215, SLC29A1, RGL2, FBXO41, CMTM7, RHPN1-AS1, CCDC74A, MINCR, VGF, DBNDD1, LINC02983, PRDM12, LOC101929536, CGREF1, DUSP14, PALD1, GALNT6, ZNF74, FAM89A, NUP50-DT, MSANTD3, MORC4, DIMT1, JPH1, RIPK2, LYAR, MTERF3, POP1, PRDX4, CCT4, ODC1, RAN, CCT2, PSAT1, KCTD14, TYRO3, CCDC157, ELFN1-AS1, ASIC1, METTL1, CDK4, SNAI1, ZFAND2A-DT, OVGP1, LINC01356, CCDC113, TCTN1, TIGD4, MELTF-AS1, WDR97, PDZD7, EVX1-AS, LCN12, TEX45, SCX, RHPN1, KIFC2, CPNE7, MLXIPL, LMTK3, RHBDL1, NLE1, ALKBH2, C19orf48, PUS1, NOP56, SOX12, TMEM231, FAM241B, NOB1, ZNF579, PTOV1, FXYD5, ASPHD1, SAPCD2, HES6, ANKRD13B, DHCR7, RECQL4, BOP1, TRIB3, SRCIN1, LARGE2, PTP4A3, GTF2IRD1, SLC3A2, PRELID3A, REM2, FAM222A-AS1, MYC, ANAPC7, POLR1G, EPOP, DDN, SLC7A5, VPS9D1-AS1, NDUFAF8, LOC100131785, SIK1, LRRC43, KISS1, CEBPB, NPEPL1, LOC124904942, UCKL1, KLHL35, ARPC1B, PKD1-AS1, GDF15, PDCD2L, NME1, CITED4, PPP1R35, MILIP, JMJD4, HYAL3, EXOSC4, PYCR3, GALK1, PAFAH1B3, EEF1AKMT4, NAA10, NXT1, ATP6V1F, BID, XDH, SPINK2, SCIN, CASP5, CBX2, DANCR, SNHG7, B9D1, AEN, SNHG8, NMB, CDC45, CDCA4, ITM2C, TMEM132A, DDIT4, TFR2, NECTIN4, CCNI2, C3orf33, GAS5, RPL12, GZMA, S100P, IER3, HOXC11, UPK2, SFTA2, VENTX, FGF18, SMTNL2, SPDYC, PRR36, GRHL3, PRR7, CCDC85B, NDUFA11, SELENOH, IER5L, H2AC25, PAQR4, TMEM191A, GNB1L, PYCR1, CHCHD6, KLC3, AMH, RTN4RL2, DUSP9


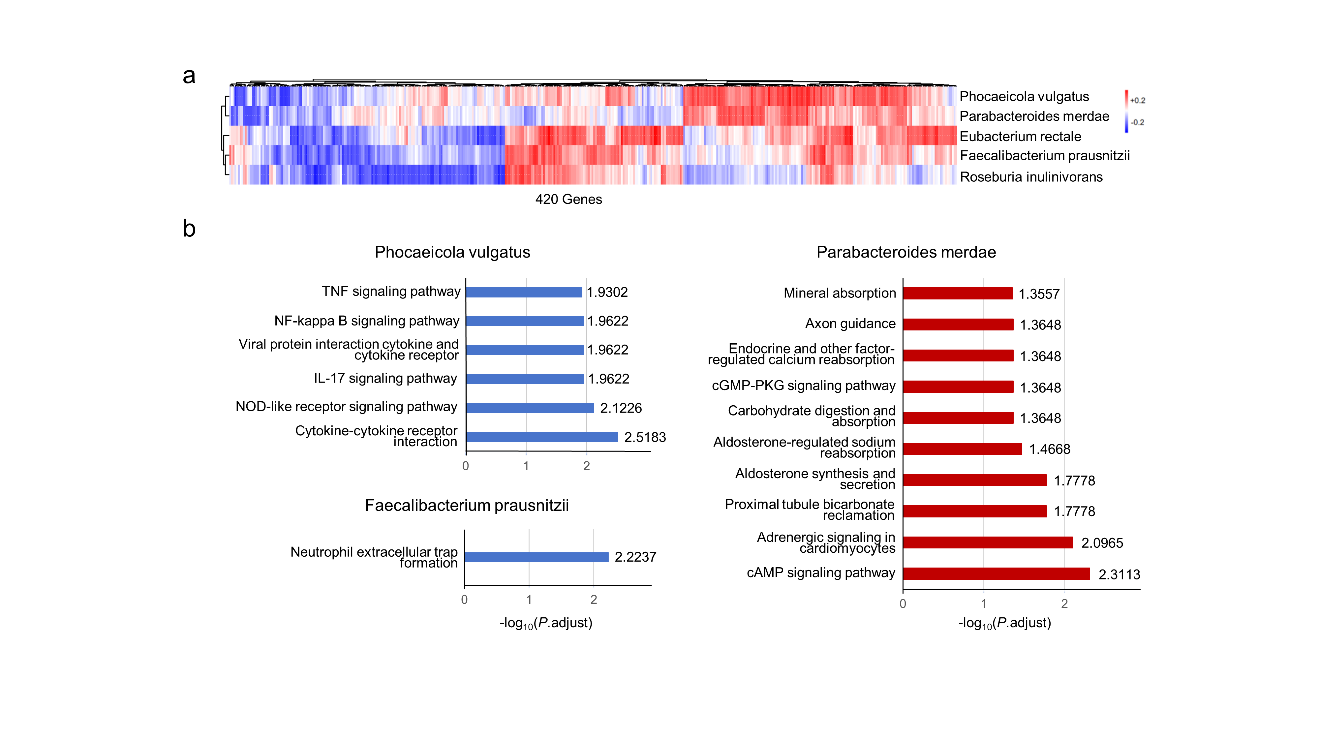


**Figure S2 The association between the first five microbial species with high confidence from MICAH and host gene expression. a.** Correlation between the abundance of identified species and the expression of DEGs for top five species. Each row is a species and each column is a gene. **b.** Enriched pathways of correlated genes of three species, and the other two with no significant pathways.


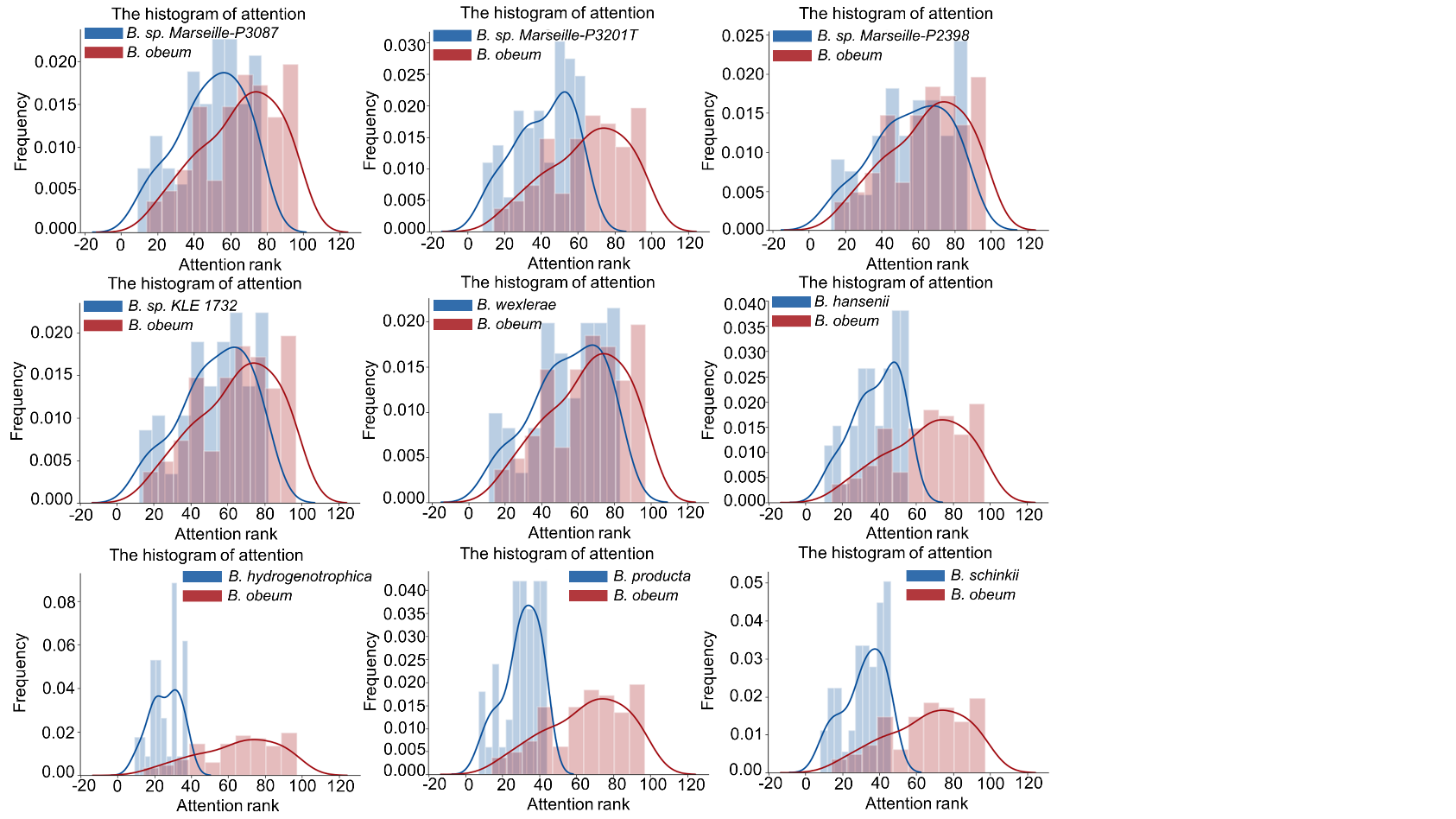


**Figure S3 The distribution of attention score rank of *Blautia obeum* and other species belonging to Blautia in COAD samples.** The Wasserstein distances between the distributions corresponding to the above figure are 13.8590, 21.4923, 8.0341, 9.7831, 9.1786, 38.7949, 33.7111, 25.4211, 32.3830.


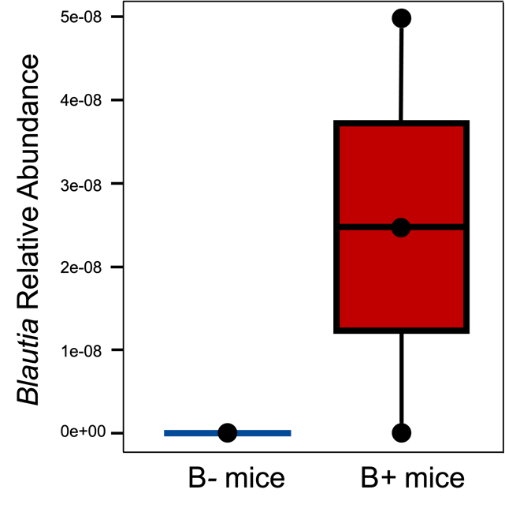


**Figure S4 Relative abundance of *Blautia* in tumor tissues.** The *Blautia* RNA in the tumor tissues in B*-* mice (n=3) and B*+* mice (n=3) were quantified as relative abundance. Black dot: individual mouse. Blue and red box: mean ± interquartile range (25th to 75th percentile) of B*-* and B+ mice, respectively.


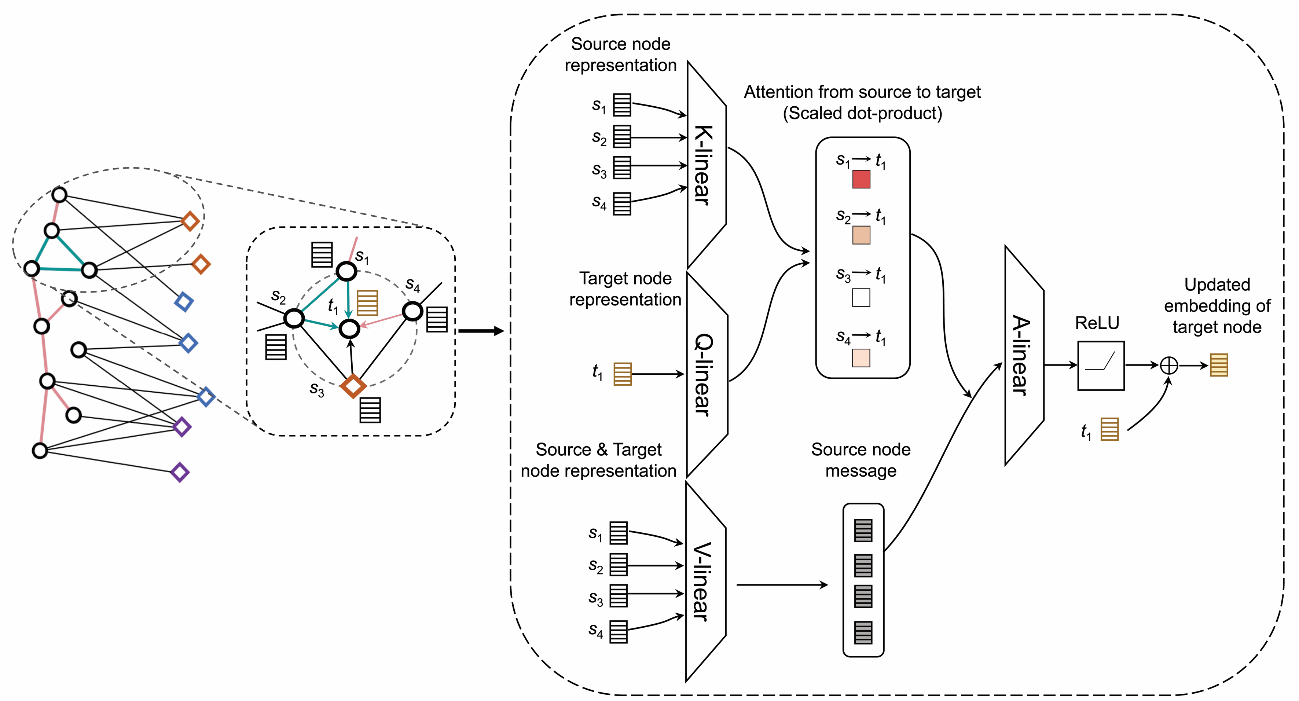


**Figure S5** **Details of attention mechanism for node embedding update.** When we update node embedding, each node is regarded as a target node, and the information of neighbor nodes is used to update the target node embedding. Using a species node $t_{1}$ as the target node for illustration. Its neighbor nodes, $s_{1}$, $s_{2}$,$s_{3}$, and $s_{4}$, are considered as source nodes while updating the embedding of the target node. We use node-type-dependent linear projection functions, ${K\_linear}_{\tau(s_{i})} (i=1, 2, 3, 4)$ and ${Q\_linear}_{\tau(t_{1})}$, to map the embedding of source nodes and the target node, respectively, obtaining key vectors and a query vector. Here, $\tau\left( * \right)$ indicates the type of a given node. Then, the similarity between each key vector and the query vector is calculated as an attention score from the source node to the target node. Meanwhile, we use node-type-dependent linear projection functions, ${V\_linear}_{\tau(s_{i})}$, to map the embedding of all source nodes, obtaining value vectors as a message of each source node to the target node. Next, the messages from all source nodes are aggregated and weighted by the corresponding attention scores. By integrating the original node embedding of the target node with the aggregated node messages, we can obtain an updated embedding of the target node.

**Table S1. The reproducibility performance on the five fungi datasets**

| Batch | Data | MICAH | PopPhy-CNN | Netmoss | SVM | RF | Enet | Lasso | MIIDL |
| --- | --- | --- | --- | --- | --- | --- | --- | --- | --- |
| Batch_1 | 80% | 0.3907 | 0.3510 | 0.2776 | 0.2593 | 0.3314 | 0.2315 | 0.2251 | 0.1814 |
|  | 85% | 0.3826 | 0.3721 | 0.3438 | 0.2933 | 0.3570 | 0.2421 | 0.2794 | 0.1753 |
|  | 90% | 0.3954 | 0.3758 | 0.3785 | 0.3128 | 0.3849 | 0.3242 | 0.3469 | 0.2221 |
|  | 95% | 0.5285 | 0.3958 | 0.4056 | 0.3691 | 0.4594 | 0.3893 | 0.4213 | 0.2963 |
| Batch_2 | 80% | 0.5834 | 0.3653 | 0.2689 | 0.3854 | 0.5206 | 0.2405 | 0.2593 | 0.2943 |
|  | 85% | 0.5873 | 0.3925 | 0.3141 | 0.3305 | 0.5282 | 0.3285 | 0.2948 | 0.3085 |
|  | 90% | 0.5925 | 0.3374 | 0.3464 | 0.4092 | 0.5709 | 0.3173 | 0.3384 | 0.3278 |
|  | 95% | 0.6175 | 0.3953 | 0.4545 | 0.4101 | 0.6002 | 0.3979 | 0.3523 | 0.3503 |
| Batch_3 | 80% | 0.4381 | 0.3192 | 0.2935 | 0.2477 | 0.3783 | 0.2850 | 0.3625 | 0.1803 |
|  | 85% | 0.4588 | 0.3509 | 0.3278 | 0.2644 | 0.4291 | 0.3792 | 0.3491 | 0.1699 |
|  | 90% | 0.4630 | 0.3435 | 0.3572 | 0.3630 | 0.4179 | 0.3526 | 0.3582 | 0.2262 |
|  | 95% | 0.4699 | 0.4369 | 0.4544 | 0.4318 | 0.4665 | 0.4295 | 0.3102 | 0.3570 |
| Batch_4 | 80% | 0.4302 | 0.2970 | 0.2923 | 0.1932 | 0.3783 | 0.2891 | 0.2445 | 0.2293 |
|  | 85% | 0.4469 | 0.3135 | 0.3032 | 0.2580 | 0.4452 | 0.2993 | 0.2454 | 0.2473 |
|  | 90% | 0.4879 | 0.3756 | 0.3309 | 0.2616 | 0.4888 | 0.3107 | 0.2951 | 0.2590 |
|  | 95% | 0.4940 | 0.4576 | 0.3403 | 0.3269 | 0.4913 | 0.3606 | 0.3699 | 0.3431 |
| Batch_5 | 80% | 0.4348 | 0.4044 | 0.2387 | 0.1880 | 0.3714 | 0.3305 | 0.3379 | 0.2919 |
|  | 85% | 0.4527 | 0.4129 | 0.2602 | 0.2327 | 0.4213 | 0.3406 | 0.3624 | 0.2903 |
|  | 90% | 0.4821 | 0.4472 | 0.3151 | 0.2448 | 0.4521 | 0.3659 | 0.3905 | 0.3303 |
|  | 95% | 0.5929 | 0.4971 | 0.3807 | 0.3330 | 0.4558 | 0.3759 | 0.4273 | 0.3415 |

**Table S2. The microbial communities identified by MICAH**

| Cancer | NCBI Taxonomy ID |
| --- | --- |
| COAD | 853，821，360807，46503，39491，204516，28118，88431，410072，40520，1450439，592978，46506，818，47678，357276，817，338188，166486，39488，208479，46228，154046，28116，28117，301302，387661，658082，28111，29361，328814，1512，820，39486，35833，246787，544645，860，33038，33039，310298，329854，39496，39485，384638，823，214851，74426，1519439，387090，106588，487174，310297，61171，712288，1531，333367，376805，45851，1735，457416，165179，169435，936562，1322，239935，1535，102148，39778，290053，29347，450749，729，29466，851，1871013，168384，371601 |
| ESCA | 28132，1161412，563031，712461，76122，425941，29466，706435，60133，28137，341694，1852368，28131，1313，860，1660，712469，712288，28037，187326，851，218538，796942，665939，936562，1303，544580，1019，712976，28135 |
| HNSC | 563031，28132，1161412，60133，712461，28137，425941，936562，851，28131，712976，76122，712288，706435，341694，467210，282402，671230，33033，218538，28135，936595，28125，729，158，712357，29466，1852365，189722，187326，1859694，1328，1852368，1303，713008，39777，712469，28129，824，386414，28130，69823，1318，837，40542，43995，419005，1019，28127，39950，28126，712991，457416，242750，1660，1261，118748，28134，796942，35517，1321783，1382，53418，1739543，35519，31973，665939，936375，157691，1588755，1795832，157688，854，46124，1305，2124，135083 |
| READ | 821，853，28118，46503，39491，40520，360807，39488，818，47678，1450439，592978，357276，301302，487174，817，820，28111，88431，28117，46506，166486，46228，154046，35833，204516，328814，33033，208479，39486，544645，1739279，39485，29361，410072，387661，74426，338188，165179，33038，246787，310298，1512，384638，936561，341694，28116，936562，860，1519439，658082，823，214851，712288，102148，851，329854，310297，106588，39950，1739435，827，45851，76122，169435，1535，333367，39492，29347，33039，1531，712359，1917878，1871013，1322，100886，1735，61171，376805 |
| STAD | 563031，60133，76122，1161412，28132，712461，28131，210，425941，29466，729，712976，28137，1596，341694，1859694，1313，187326，28037，1852368，39950，706435，712288，282402，712469，1303，467210，1608882，28135，69823，851，860，39777，665939，218538，1739543，33033，796942，1328，40542，29391，1715211，157688，1633，936595，1583098，936563，1624，712991，1318，45634，936562，419005，28125，1581071，28130，46124，739，39778，157691，33959 |

**Table S3. Intratumoral microbial species associated with the five cancer types from Peryton**

| Cancer | NCBI Taxonomy ID |
| --- | --- |
| COAD | 1019，102148，1261，1305，1328，135083，137732，1383，143361，150055，157688，158787，1598，1624，172042，181487，199，200，204，239935，28112，28123，28124，28126，28127，28131，28133，28135，281920，29391，29466，310297，316，33033，33038，33039，33959，341694，35519，39950，40520，40542，419015，425941，46124，47678，53419，573，61592，69823，729，739，76122，817，820，821，82203，823，824，827，84109，84112，84135，851，853，859，860，97478，166486 |
| ESCA | 851，28132 |
| HNSC | 1613，712117，490，860，425941，69823，851，40542，157688，109328，33033，1309 |
| READ | 1584，28132，817，820 |
| STAD | 851，210 |

**Table S4. Body site attribution for cancer types from Sourcetracker2.**

| **Cancer type** | **COAD** | **HNSC** | **STAD** | **READ** |
| --- | --- | --- | --- | --- |
| cecum | 0.0795 | 0.0002 | 0.0003 | 0.0611 |
| colon | 0.0982 | 0.0007 | 0.0003 | 0.0405 |
| rectum | 0.0838 | 0.0052 | 0.0042 | 0.0941 |
| duodenum | 0.0082 | 0.1142 | 0.0458 | 0.0085 |
| blood | 0.0027 | 0.0046 | 0.0028 | 0.003 |
| ear | 0.0013 | 0.0064 | 0.001 | 0.0043 |
| esophagus | 0.0077 | 0.1162 | 0.1122 | 0.0025 |
| lung | 0.0033 | 0.0082 | 0.0063 | 0.0037 |
| nose | 0.0056 | 0.002 | 0.0033 | 0.003 |
| oral | 0.014 | 0.0781 | 0.069 | 0.0031 |
| skin | 0.0004 | 0.0008 | 0.0004 | 0.0007 |
| stomach | 0.0034 | 0.0121 | 0.1624 | 0.0033 |
| trachea | 0.0034 | 0.0273 | 0.0473 | 0.0019 |
| Unknown | 0.6885 | 0.624 | 0.5447 | 0.7703 |

**Table S5. The species contributed by attention and both phylogenetic and metabolic relationships in MICAH**

| ***Attention*** | ***Phylogenetic and metabolic relationships*** |
| --- | --- |
| *[Eubacterium] rectale* | *[Clostridium] symbiosum* |
| *Dorea longicatena* | *Bacteroides cellulosilyticus* |
| *Blautia obeum* | *Lachnospira eligens* |
| *Oscillibacter sp. ER4* | *[Bacteroides] pectinophilus* |
| *Bacteroides thetaiotaomicron* | *Subdoligranulum variabile* |
| *Phocaeicola dorei* | *Collinsella aerofaciens* |
| *Anaerobutyricum hallii* | *Pseudoflavonifractor capillosus* |
| *Roseburia faecis* | *Phocaeicola plebeius* |
| *Hungatella hathewayi* | *Enterocloster asparagiformis* |
| *Roseburia intestinalis* | *Phocaeicola salanitronis* |
| *[Ruminococcus] lactaris* | *Butyrivibrio crossotus* |
| *Parabacteroides johnsonii* | *Holdemanella biformis* |
| *Fusobacterium periodonticum* | *Anaerotruncus colihominis* |
| *Bacteroides eggerthii* | *Fusobacterium sp. CM21* |
| *Fusobacterium nucleatum* | *[Clostridium] leptum* |
| *Phocaeicola coprocola* | *Solobacterium moorei* |
| *Butyricimonas virosa* | *[Clostridium] scindens* |
| *Bilophila wadsworthia* | *Marvinbryantia formatexigens* |
| *Phocaeicola coprophilus* |  |
| *Barnesiella intestinihominis* |  |
| *Fusobacterium sp. oral taxon 370* |  |
| *Veillonella sp. 3_1_44* |  |
| *Haemophilus parainfluenzae* |  |
| *Prevotella copri* |  |

**Table S6. Main effects on tumor volume before the immunotherapy**

| **Effects** | **DF** | **SS** | **MS** | **F value** | **P value** |
| --- | --- | --- | --- | --- | --- |
| *B. massiliensis* | 1 | 1679 | 1679 | 0.776 | 0.389 |
| Time | 3 | 141601 | 47200 | 46.448 | 1.2e-15*** |
| *B. massiliensis**time | 3 | 1776 | 592 | 0.583 | 0.629 |

Two-way repeated measures analysis of variance (ANOVA) for pre-immunotherapy was performed. *B. massiliensis* supplement and time points are considered categorical variables. ***P<0.001; *B. massiliensis**time, interaction of *B. massiliensis* supplement and time; DF, degree of freedom; SS, sum of squares; MS, mean squares; *B. massiliensis, Blautia massiliensims*.

**Table S7. Longitudinal mixed-effects model post-immunotherapy**

| **Coefficients** | **Estimate** | **Standard Error** | **T value** | **P value** |
| --- | --- | --- | --- | --- |
| Intercept | 95.7400 | 65.2242 | 1.468 | 0.1474 |
| B+_c | -3.6937 | 96.7431 | -0.038 | 0.9697 |
| B-_t | -0.9714 | 92.2409 | -0.011 | 0.9916 |
| B+_t | 14.1511 | 103.1285 | 0.137 | 0.8913 |
| Day 12 | 29.3878 | 75.6691 | 0.388 | 0.6990 |
| Day 15 | 95.2236 | 75.6691 | 1.258 | 0.2125 |
| Day 18 | 144.4445 | 75.6691 | 1.909 | 0.0605 |
| Day 21 | 439.3625 | 75.6691 | 5.806 | 1.85e-07*** |
| B+_c*Day 12 | -2.7360 | 112.2354 | -0.024 | 0.9806 |
| B-_t*Day 12 | 24.5740 | 107.0123 | 0.230 | 0.8191 |
| B+_t*Day 12 | -54.8854 | 119.6434 | -0.459 | 0.6479 |
| B+_c*Day 15 | 46.5306 | 112.2354 | 0.415 | 0.6798 |
| B-_t* Day 15 | -54.6315 | 107.0123 | -0.511 | 0.6113 |
| B+_t*Day 15 | -178.5857 | 119.6434 | -1.493 | 0.1402 |
| B+_c*Day 18 | 5.5235 | 112.2354 | 0.049 | 0.9609 |
| B-_t* Day 18 | -103.3853 | 107.0123 | -0.966 | 0.3374 |
| B+_t*Day 18 | -246.6050 | 119.6434 | -2.061 | 0.0431* |
| B+_c*Day 21 | -76.4617 | 112.2354 | -0.681 | 0.4980 |
| B-_t* Day 21 | -263.9626 | 107.0123 | -2.467 | 0.0162* |
| B+_t*Day 21 | -549.2536 | 119.6434 | -4.591 | 1.96e-05*** |
| **Contrasts** | **Estimate** | **Standard error** | **Z value** | **P value** |
| B+_t v.s. B-_t | -270.17 | 103.13 | -2.620 | 0.0175* |
| B+_c v.s. B-_c | -80.16 | 96.74 | -0.829 | 0.6488 |

A longitudinal mixed-effects model was built to estimate the effects of experimental conditions (n=4-6 mice/group), time, and the interaction between experimental conditions and time on tumor volume. The Satterthwaite approximation produced P values with Sidak’s correction for multiple comparisons. *P<0.05, ***P<0.001; B+_c, B+ mice without treatment; B-_t, B- mice with anti-PD1; B+_t, B+ mice with anti-PD1.

**Table S8. ANOVA table of the Longitudinal mixed-effects model post-immunotherapy**

| **Effects** | **DF** | **SS** | **MS** | **F value** | **P value** |
| --- | --- | --- | --- | --- | --- |
| Conditions | 3 | 531944 | 177315 | 3.01 | 0.0591 |
| Time | 4 | 755982 | 188996 | 11.003 | 6.13e-07*** |
| Conditions*time | 12 | 539673 | 44973 | 2.618 | 0.006125** |

ANOVA table of longitudinal mixed-effects model to show the main effects of experimental conditions after immunotherapy started. The experimental conditions included B+ mice with anti-PD1, B- mice with anti-PD1, B+ mice without treatment, and B- mice without treatment. **P<0.01, ***P<0.001.

**Table S9. The number of samples of the five cancer types from TCMA**

| Cacner type | COAD | ESCA | HNSC | READ | STAD |
| --- | --- | --- | --- | --- | --- |
| The number of primary tumor | 125 | 60 | 155 | 45 | 127 |

**Supplementary Section S1. The construction of phylogenetic links at different phylogenetic levels.**

We considered the phylogenetic relationships that two species at different phylogenetic levels. We constructed the phylogenetic relation matrix, $D_{M\times M}^{2}$, in which $d_{i_{1}i_{2}}^{2}=1$ if the ${i_{1}}^{th}$ species and the ${i_{2}}^{th}$ species ($i_{1}\neq i_{2}$) are in the same genus/family/order/class/ phylum; otherwise, $d_{i_{1}i_{2}}^{2}=0$. Based on these phylogenetic relation matrices, we identified cancer-associated microbial species, respectively.

We found using genus level to construct microbe-microbe relationships could acquire more intratumoral microbiome associated with COAD. Among these 40 species uniquely identified by MICAH using genus-level links, there are six species (15%) experimentally supported related to COAD, which is the highest than these at other phylogenetic levels. Therefore, we finally selected genus levels to construct heterogeneous graph and capture the relationships between intratumoral microbes and cancer tissues.

**Supplementary Section S2. Determining the *p*-value for a species to a cancer type.**

Our aim is to identify the microbial community associated with a certain cancer type by detecting species with consistently high contributions to samples with the cancer type. Based on the step introduced in the main text, we have obtained a species set for each sample in which each species highly contributes to the sample. In this section, we illustrated how to determine species with consistently high contributions to samples with a cancer type using the $c^{th}$ cancer type as an example.

Suppose there are $U_{c}$ samples with the $c^{th}$ cancer type, denoted as $P_{1},P_{2},\cdots,P_{U_{c}}$ respectively. For each sample, we have obtained a set of species with high contributions, denoted as $S_{1},S_{2},\cdots,S_{U_{c}}$. Intuitively, the microbial species associated with the $c^{th}$ cancer type would be $S_{1}\cup S_{2}\cup\cdots\cup S_{U_{c}}$. However, there may be random factors in the selection of the species associated with a sample, which will greatly increase the false positives. To mitigate this effect, we use a statistical method to select statistically significant species for cancer-associated microbial communities. Specifically, we consider the problem into the classic ball-drawing problem: *i*) Choose a number randomly from the set $S=\{{|S}_{1}|,{|S}_{2}|,\cdots,{|S}_{U_{c}}|\}$, denoted as ${|S}_{z}| (z\in\{1,2,\cdots, U_{c}\})$. Then, remove it from $S$. *ii*) Draw ${|S}_{z}|$balls at once from a bag with $M$ different balls. Record the ${|S}_{z}|$ balls and then put them back. We repeat these two processes until $S=\emptyset$. It is important to note that we determine the number of balls selected in each experiment successively, but the order of ${|S}_{z}|$ should have no effect. That is to say, drawing ${|S}_{i}|$ or ${|S}_{j}|$ balls first are equivalent ($1\leq i\neq j\leq U_{c}$). In this case, the probability of drawing a certain number of balls $t$ or more times can be calculated, corresponding to a species highly contributing to $t$ or more samples with the $c^{th}$ cancer type. If the cumulative probability is less than 0.05, we consider the species to be significantly associated with the cancer type.

However, since the $U_{c}$ experiments are not simply repeated, it is extremely computationally intensive to calculate the probability. To address this, we use the method of enlarging and reducing. We use Formula (1) to calculate the probability of a ball being selected while randomly drawing $b$ balls in a bag with $m$ balls. As $b$ increases, the value of Formula (1) increases. We use Formula (2) to calculate the probability of a ball not being selected. As $b$ increases, the value of Formula (2) decreases.

$$\begin{aligned} \frac{\left( \begin{aligned} m-1 \\ b-1 \end{aligned} \right)\text{ }}{\left( \begin{aligned} m \\ b \end{aligned} \right)}\#\left( 1 \right) \end{aligned}$$

$$\begin{aligned} \frac{\left( \begin{aligned} m-1 \\ b \end{aligned} \right)\text{ }}{\left( \begin{aligned} m \\ b \end{aligned} \right)}\#\left( 2 \right) \end{aligned}$$

Therefore, the probability of the event “the number of a ball is drawn equal to or more than $t$ times” is measured as Formula (3). This enlarging and reducing method enables us to calculate the probabilities more efficiently than directly computing them for the $U_{c}$ experiments, allowing us to identify species significantly associated with the cancer type without incurring prohibitively high computational costs.

$$\begin{aligned} P_{c}^{i}\leq\sum_{t=T_{c}^{i}}^{U_{c}} \left( \begin{aligned} U_{c} \\ t \end{aligned} \right)\times\left[ \frac{\left( \begin{aligned} M-1 \\ S_{c}^{max}-1 \end{aligned} \right)}{\left( \begin{aligned} M \\ S_{c}^{max} \end{aligned} \right)} \right]^{t}\times\left[ \frac{\left( \begin{aligned} M-1 \\ S_{c}^{min} \end{aligned} \right)}{\left( \begin{aligned} M \\ S_{c}^{min} \end{aligned} \right)} \right]^{U_{c}-t}\#\left( 3 \right) \end{aligned}$$

Where $U_{c}$ is the total number of samples with the $c^{th}$ cancer type. $M$ is the number of all species. $S_{c}^{max}=\max_{1\leq z\leq U_{c}\}} {|S}_{z}|$ and $S_{c}^{min}=\min_{1\leq z\leq U_{c}\}} {|S}_{z}|$ represent the sizes of the largest and smallest sets that significantly contribute to the corresponding samples with the $c^{th}$ cancer type that each species significantly contributed to, respectively. If the value on the right-hand side of the Formula (3) is less than 0.05, the species will be regarded as significantly contributing to the $c^{th}$ cancer type.

**Supplementary Section S3. Parameter optimization of machine learning methods.**

The selection of parameters is crucial for the performance of a data-driven model. In this study, we tuned multiple parameters for the machine learning methods. For MIIDL, we tried different parameter sets, including:

- qc: 0.0001, 0.001, 0.1, and 0.3;
- Normalization:$\log_{2} (x+1)$, $\log_{2} (x)$, $\ln x$, $\ln(x+1)$, mean, median, z-score, and none;
- Imputation: knn, mean, median, minimum, and none;
- Epoch: 100, 200, 400, and 600.

Finally, we selected the parameter combination that performed the best on the TCMA dataset, which has qc: 0.00001; normalization: loge; imputation: mean; and epoch: 600.

For the four methods (Lasso, Enet, RF, and SVM), we used the MetaML software and optimized the classifiers using GridSearchCV for the best model. Finally, the parameters used for the four methods were as follows:

- Lasso: i enet s f1_macro r 1;
- Enet: i enet s f1_macro r 1;
- RF: i enet s f1_macro r 1;
- SVM: i enet s f1_macro r 1.
